# iNaturalist and Structured Mammal Surveys Reflect Similar Species Richness but Capture Different Species Pools Across the United States

**DOI:** 10.1002/ece3.71805

**Published:** 2025-07-20

**Authors:** Daniel J. Herrera, Christopher M. Schalk, Alex J. Jensen, Benjamin R. Goldstein, Brigit R. Rooney, Roland Kays, William J. McShea, Michael V. Cove

**Affiliations:** ^1^ North Carolina Museum of Natural Sciences Raleigh North Carolina USA; ^2^ USDA Forest Service, Southern Research Station Nacogdoches Texas USA; ^3^ Department of Forestry and Environmental Resources North Carolina State University Raleigh North Carolina USA; ^4^ Smithsonian's National Zoo and Conservation Biology Institute Front Royal Virginia USA

**Keywords:** acoustic survey, biodiversity, camera trap, citizen science, mist net, survey design

## Abstract

Crowd‐sourced biodiversity data, such as those housed in the iNaturalist platform, are increasingly used to monitor species distributions. Such data represent unstructured biodiversity surveys that are generally comprised of incidental observations and do not report variation in sampling effort. These discrepancies may yield data that is incongruent with data from structured surveys. To assess whether mammalian iNaturalist data are reflective of data from traditional structured surveys, we calculated and compared measures of mammalian species richness and species pool similarity using data from unstructured surveys (i.e., iNaturalist) and data from structured camera trap surveys and bat acoustic surveys. We found that data from structured and unstructured surveys generally document similar mammalian species richness, but the two survey types document different species pools. Human population density and proxies for species pool breadth were most strongly associated with discrepancies in datasets, with data being most similar in areas of high human population density and lower species richness. Our analyses revealed that dataset similarity varied across geography and community metric for most taxa, but that structured and unstructured surveys produced consistently unreconcilable datasets for bats. These findings suggest that unstructured datasets like iNaturalist may offer reliable data for some taxa and geographies, but that these data are not universally applicable to all research scenarios.

## Graphical abstract

iNaturalist data is widely used in ecology, but is known to contain spatial and taxa‐specific biases. We compared species richness and species pools using data from iNaturalist and structured surveys (i.e., camera traps and acoustic bat surveys) across the United States. We found that iNaturalist and structured surveys generally report similar richness, but that iNaturalist reports notably different species than structured surveys—suggesting that neither method is free of error or bias.

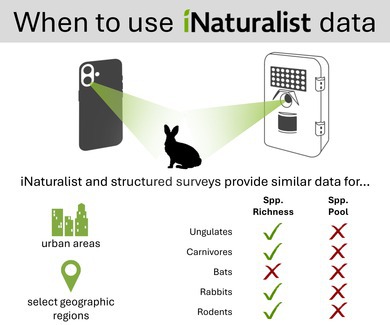

## 1 Introduction

Robust datasets guide effective ecosystem management decisions, while datasets of limited size and accuracy can lead to ineffective outcomes (Costello et al. 2013; Stephenson and Stengel 2020; Weissgold 2024). Increased sample size can often mitigate this shortcoming, but high‐quality data collection can be logistically and financially infeasible at the scale required by decision makers (Dobson et al. 2020; Kindsvater et al. 2018; Troudet et al. 2018). In response, practitioners often rely on existing datasets to inform their management actions (Dobson et al. 2020; Stephenson and Stengel 2020). In the field of wildlife ecology, crowd‐sourced datasets (also called citizen science) of species observations are especially valued since they provide information about species distributions at no cost to the data users. Although such data are inexpensive, they are often unstandardized, incomplete, and biased toward particular taxa and geographies (Dimson and Gillespie 2023; Dobson et al. 2020). Thus, decision makers must base their management on easily obtained but incomplete datasets or on more robust datasets that are limited in geographic scope and require substantial time, money, and effort to collect.

Structured survey methods are generally seen as the ‘gold standard’ since they follow clearly defined sampling protocols. Data arising from structured surveys can be summarized as detection/non‐detection data (also called “presence/absence data”) indicating whether the target organism is observed during each survey, with unobserved species resulting in putative absences. On the other hand, unstructured data are considered “presence‐only” because there is no direct record of surveys that did not produce detections of the species (Bayraktarov et al. 2019; Foody 2011; Li et al. 2011; MacKenzie 2005a, 2005b). Statistically, detection/non‐detection data can be analyzed with models that directly account for variation in the sampling process, such that remaining variation in the observed data can be attributed to the underlying ecological process. Conversely, models of presence‐only data depend on extra assumptions about sampling effort (Foody 2011; Phillips et al. 2006). Presence‐only data are often comprised of incidental observations, and observers may elect to report only select sightings (Jessup 2003; MacKenzie 2005a, 2005b). However, such variation in effort is unobserved and is challenging to control for during analysis.

Despite the shortcomings of unstructured survey data, the sheer abundance, low cost, low effort, and wide geographic range of these data are particularly attractive to researchers (Dobson et al. 2020; López‐Guillén et al. 2024; Wahlberg et al. 2023). The iNaturalist platform in particular has enjoyed immense popularity since its release in 2008 (López‐Guillén et al. 2024; Martínez‐Sagarra et al. 2022; Mesaglio and Callaghan 2021). iNaturalist is an online platform with two mobile applications (iNaturalist and Seek) that allow users to upload photographic or audio evidence of organisms to the iNaturalist database of observations where, depending on user preferences, it may be integrated into the Global Biodiversity Information Facility (GBIF; Altrudi 2021). Given the ease of participating in data collection, crowd‐sourced data has become immensely abundant in recent years. Indeed, the iNaturalist platform boasts over 2.9 million users and data produced by iNaturalist has been used in over 4000 peer‐reviewed publications, suggesting that the platform plays a pivotal role in conservation science (Global Biodiversity Information Facility 2024; López‐Guillén et al. 2024).

Although iNaturalist data continue to be widely used, they are known to have taxonomic and spatial biases. Observations tend to be disproportionately clustered around developed areas, trails, and roads, offering limited insights into ecological communities beyond the reach of basic human infrastructure (Backstrom et al. 2024; Di Cecco et al. 2021; Dimson and Gillespie 2023; Taraporevala et al. 2025). Within populated areas, data are concentrated in affluent areas with abundant greenspace and are not temporally consistent with observations from passive structured surveys (Carlen et al. 2024; Estien et al. 2024; Taraporevala et al. 2025). Even where observations are abundant, many observers use iNaturalist as either a means of identifying an unknown organism or as a personal species checklist, leading to observer‐specific variation in taxonomic sampling effort (Goldstein and Stoudt 2025). In either case, observers typically only upload a single observation of a species, even if the species is frequently encountered (Di Cecco et al. 2021). Finally, observations tend to be biased toward large, charismatic species that are approachable and easily photographed (Di Cecco et al. 2021; Goldstein et al. 2024; Stoudt et al. 2022).

Given the biases present in iNaturalist data, it stands to reason that these data do not consistently reflect the same biodiversity patterns as data from structured surveys. Indeed, many studies have explored the degree to which unstructured surveys like iNaturalist produce datasets that resemble those produced by structured surveys. Species richness estimates derived from unstructured surveys generally resemble those derived from structured surveys for most taxa at a coarse (i.e., 10,000‐km^2^ sampling grid) spatial scale (Daru and Rodriguez 2023). At finer scales, iNaturalist data seemingly correspond to structured survey data for species of bees (Apidae family), dragonflies (Anisoptera infraorder), and damselflies (Zygoptera suborder), but not for butterflies (Heteroneura clade; Shirey et al. 2021; Chesshire et al. 2023; Bullion and Bahlai 2024). Reptile observations on iNaturalist differ from structured survey data (Forti et al. 2024), whereas the fidelity of amphibian records remains largely unexplored (Oliver et al. 2025). iNaturalist records of birds disproportionately report large‐bodied and flocking birds, but similarity between unstructured and structured datasets tends to improve with increasing iNaturalist observations (Callaghan et al. 2021; Jacobs and Zipf 2017). Mammal detections on iNaturalist are comparable to structured surveys of deceased specimens (i.e., roadkill; Périquet et al. 2018). However, data from structured and unstructured surveys of live marine mammals differ (Viola et al. 2024), and such an analysis of live terrestrial mammals has not yet been undertaken.

In this study, we explored the factors contributing to the comparability of iNaturalist mammal observations and data from structured mammal surveys across the contiguous United States (US). Furthermore, we assessed if iNaturalist is an appropriate alternative for structured surveys in the context of two community metrics: similarity in species richness and similarity in species pools. We hypothesized that the similarity of mammalian communities reported by iNaturalist and structured surveys would vary spatially due to ecological variables and differences in survey effort. Specifically, we predicted that iNaturalist observations and data from structured surveys would yield similar estimates of species richness and species pools and that similarity between datasets would be positively associated with the prevalence of protected lands and the ease of traversing the landscape (i.e., less rugged landscapes), since humans are more likely to access and document species in these areas (Mair and Ruete 2016). We also predicted that similarity between datasets would be positively associated with human population density, but that the effect of humans diminishes in the most heavily urbanized areas (i.e., the effect of human population density is quadratic). Finally, we predicted that these metrics would be most similar in areas with fewer species and where survey effort is substantial, since both factors have the potential to produce more complete datasets. If iNaturalist data accurately reflects community composition, these data may offer reasonable insight into community dynamics in regions where structured surveys have not occurred. The prudent use of iNaturalist data to understand mammalian communities may thus supplement professional wildlife surveys and contribute to the efficient distribution of limited resources in conservation.

## 2 Methods

### 2.1 Landscape Data

Previous research on the comparability of structured and unstructured survey data was conducted at a global scale of 100 × 100 km (Daru and Rodriguez 2023). We sought to use a spatial scale finer than 10,000 km^2^, but still large enough to capture spatially coincident observations from structured and unstructured surveys. We superimposed a 25 × 25 km grid over the contiguous US and removed any cell with > 10% water. We then measured the latitude and longitude of the center of each remaining cell and summarized the human population density, terrain ruggedness, and proportion of protected lands (e.g., park, conservation area, wildlife refuge, etc.) within each cell using the Gridded Population of the World dataset (resolution = 30 arc‐seconds; NASA Socioeconomic Data and Applications Center 2018), the Global Terrain Ruggedness dataset (resolution = 1‐km^2^; Carter 2018), and the PAD‐US dataset (scale = 1:24,000; United States Geological Survey 2024), respectively. For human population and terrain ruggedness, we used the mean value of the raster cells that overlapped the sampling grid cell as the final value for each variable. For the proportion of protected lands, we divided the area of protected land within each cell by the cell's total area. All variables were assessed for collinearity using Pearson's correlation coefficient (*p* ≤ 0.7) and were z‐scaled prior to analysis.

### 2.2 Standardized Survey Data

Standardized survey data for terrestrial mammals were obtained from the SNAPSHOT USA database from 2021 (Shamon et al. 2024) and 2022 (Rooney et al. 2025) and were supplemented with other publicly available camera trapping projects within the contiguous US hosted on the Wildlife Insights platform (www.wildlifeinsights.org; see Data S1 for a list of projects used). While nuances exist between the protocols of these surveys, each survey consisted of un‐baited motion‐activated camera traps aimed at game trails that were active 24‐h per day throughout their sampling period. Photographs from each camera were loaded into the Wildlife Insights online repository and were identified to the lowest taxonomy possible. After review, we excluded all observations of humans, livestock, and any animal not able to be identified to species. Additionally, we removed observations of terrestrial animals weighing less than 111.9 g—the weight of the Eastern chipmunk ( *Tamias striatus* ), which is the smallest animal that is reliably captured within our dataset (Kays et al. 2022; Wilman et al. 2014).

Bats (Chiroptera order) are not reliably detected or identifiable using camera traps. Instead, we downloaded standardized bat survey data from NABat (www.nabatmonitoring.org/). Specifically, we downloaded freely available bat data from acoustic surveys, as well as from acoustic surveys that approved our request for their embargoed data within 1 week of our request. A complete list of NABat projects that contributed data to this analysis is available in Data S1. The NABat protocol uses a generalized random‐tessellation stratified grid (10 × 10 km grid cells) across the US, and cells are selected to be sampled based on a geographic representation and ability to access the land. Within each NABat cell, surveyors conducted repeated acoustic surveys (Loeb et al. 2015). Acoustic recorders were calibrated prior to use, and bat calls were identified to species using specialized software (Data S1). Similar to the camera trap dataset, we used structured bat survey data from the years 2021 and 2022.

Prior to analysis, all species names from structured surveys were compared against species names listed in the iNaturalist database. Because iNaturalist uses the most recent taxonomy available, we settled all cases of conflicting taxonomy by renaming species according to their current name in the iNaturalist database.

### 2.3 iNaturalist Data

We batch‐exported data from iNaturalist.org in January 2023 by filtering for mammals in North America from January 1, 2021, to December 31, 2022. We then removed 14 duplicate observations with matching observation identification numbers and all observations that were not research grade or were not identified to species. Finally, we identified and removed observations based on animal sign (e.g., scat, tracks, etc.) because these types of observations are more likely to be misidentified compared to direct observations of animals (Morin et al. 2016; Spitzer et al. 2019). To remove observations of sign, we excluded observations with values in the ‘sign and song’ and ‘tracks’ columns, observations annotated as tracks or scat, and observations within the North American Animal Tracks Database, which is a project within iNaturalist that contains observations that are signs (not just tracks). For consistency between datasets, we removed all non‐Chiropteran species weighing < 111.9 g from the iNaturalist dataset, and observations of humans and livestock were automatically omitted since these species cannot achieve “research grade” in iNaturalist.

### 2.4 Controlling for Survey Effort

**FIGURE 1:**
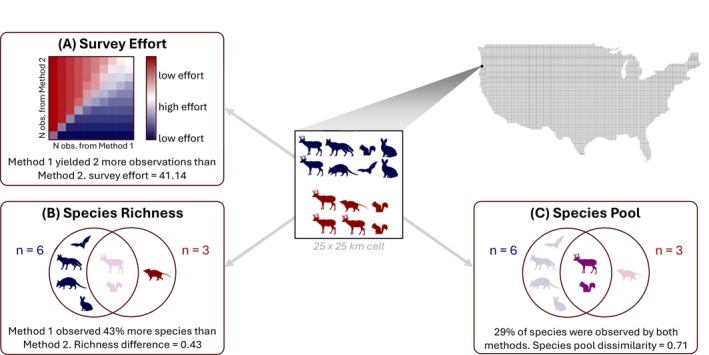
Conceptual illustration of the calculation of (A) survey effort, (B) difference in species richness, and (C) difference in the observed species pools, based on data from both survey methods in each grid cell. Animals detected in each survey method are depicted in blue or red, while individuals detected using both survey methods are depicted in purple.

We measured survey effort using the number of observations present in both the structured and unstructured datasets in each grid cell. We assumed that datasets would be most similar when they were derived from surveys of similar sampling intensity, since disproportionately larger surveys are likely to capture more varied species from sampling bias alone. We also assumed that greater volumes of data would yield more similar datasets since they would reflect more complete samples. To account for the sampling intensity of either dataset and the similarity between sampling intensities, we derived a measure of composite effort at grid cell *i*, $e_{i}$, based on the number of observations from structured, $e_{s}$, and unstructured surveys, $e_{u}$. Composite effort was based on the total volume of data in each cell, $v_{i}$, moderated by the proportional difference in the number of observations between survey methods (Figure 1A). This yields larger values when both survey methods contain many samples, moderate values when only one method has many samples, and small values when both methods have few samples.

(1) $$v_{i}=e_{s}\times e_{u}$$

(2) $$e_{i}=v_{i}\times \left(1-\frac{\left|e_{s}-e_{u}\right|}{e_{s}+e_{u}}\right)$$

### 2.5 Community Metrics

We calculated the difference in reported species richness at grid cell *i*, $N_{i},$ by subtracting the richness reported in structured data, $n_{s}$, from the richness reported in unstructured survey data, $n_{u}$, and dividing the difference by the total number of unique species detected across both methods, $n_{t}$ (Figure 1B).

(3) $$N_{i}=\frac{n_{u}-n_{s}}{n_{t}}$$

This resulted in a metric whose value is zero if both survey methods report the same number of species. Alternatively, the metric is positive if unstructured surveys reported more species but is negative if structured surveys reported more species.

Although species richness offers some insight into community composition, it can yield misleading results since two completely different species pools can produce identical richness values. We used the Jaccard dissimilarity index to assess the similarity of the species pools reported by each survey method for each grid cell (Figure 1C). The Jaccard dissimilarity index considers if a species is reported in both datasets and produces cell‐specific values that approach zero when species pools in each dataset are similar and approach one when species pools are dissimilar. Thus, values closer to zero indicate similarity between structured and unstructured datasets across both metrics employed in this study. Jaccard indices were calculated using the Vegan package (Oksanen et al. 2022).

To account for taxa‐specific variation, we calculated each of the previously described community metrics and survey effort separately for the following orders: Artiodactyla, Carnivora, Chiroptera, Lagomorpha, and Rodentia. While Cingulata and Didelphimorphia were documented in both structured and unstructured surveys, the paucity of species from these orders within the US (*n* = 1 species per order) prompted us to exclude them from order‐level analysis. Finally, we calculated each community metric for Mammalia using data from all the previously listed orders of mammals, including Cingulata and Didelphimorphia, despite not being individually analyzed.

### 2.6 Statistical Analysis

We fit a linear regression model to assess the relationship between landscape variables and the proportional difference in species richness (Equation 4). Each model included latitude, longitude, proportion of protected lands, landscape ruggedness, and survey effort as linear terms. Each model also included human population density as a quadratic term.

(4) $$y_{i}=\beta_{0}+\sum \beta_{n}x_{i}$$

We used beta regression to assess the relationship between our variables and Jaccard dissimilarity, where the distribution's shape parameters, alpha and beta, are derived from the estimated output of the previously described linear regression model and a separately estimated term, phi (Equations (5), (6), (7), (8)). Beta regression is appropriate for data between zero and one, but not for data equal to zero or one exactly; thus, we replaced Jaccard values equal to zero with values of 0.001, and likewise replaced similarity indices equal to one with values of 0.999.

(5) $$y_{i}\sim \text{Beta}\left(\alpha_{i},\beta_{i}\right)$$

(6) $$\alpha_{i}=\mu_{i}\times \varphi$$

(7) $$\beta_{i}=\left(1-\mu_{i}\right)\times \varphi$$

(8) $$\text{logit}\left(\mu_{i}\right)=\beta_{0}+\sum \beta_{n}x_{i}$$

All models were fit in a Bayesian framework using the Nimble package (de Valpine et al. 2017). Parameters representing the effects of independent variables were assigned normal priors with a mean of zero and standard deviation of 100. The parameters required by the probability distribution but not of ecological significance (i.e., phi) were instead given uniform priors constrained between zero and one hundred and zero and ten, respectively. MCMC chains consisted of 100,000 iterations with a burn‐in period of 50,000 iterations. Gelman‐Rubin statistics and trace plots were inspected for each chain to ensure convergence. Likewise, posterior predictive plots were visually inspected for each model to ensure goodness of fit. We confirmed that MCMC chains adequately mixed when Gelman‐Rubin statistics had a value ≤ 1, and trace plots showed chains consistently folding over one another. Posterior predictive plots indicated adequate model fit when datasets generated using the fitted model generally matched the observed dataset. Trace plots and posterior predictive plots are available in Data S2.

### 2.7 National Predictions

We used the estimated coefficients for each variable and the previously described sampling grid to forecast each dependent variable across the contiguous US. We held survey effort at its mean value across the sample grid since calculation of effort requires standardized survey data, which is not available in all grid cells. We then calculated the proportion of cells whose predicted community metric suggested that iNaturalist data would differ from structured survey data by ≤ 15% if structured survey data had existed in that grid cell.

## 3 Results

### 3.1 Survey Results

**FIGURE 2:**
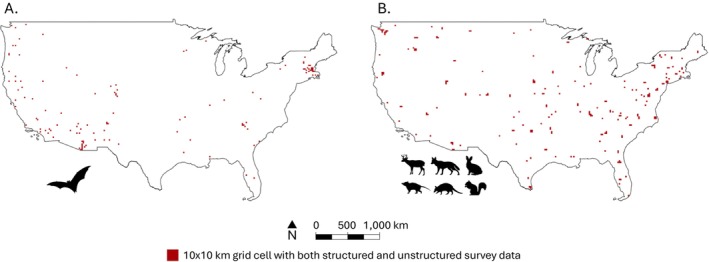
Sampling grids that contained both structured and unstructured data for (A) Chiroptera and (B) all other mammal species.

A total of 170 species were detected across 916 grid cells (Figure 2). Species‐specific summary statistics are available in Data S3. On average, each order was observed across 155 (SD = 41.35) grid cells. Across metrics, a value of zero indicates that the two survey methods produce comparable datasets. For species richness, negative values indicate that structured surveys report greater species richness than unstructured surveys. Likewise, if values are positive, unstructured surveys reported greater species richness than structured surveys. Observed species richness was most similar in Rodentia (mean = −0.01, SD = 0.21) and most dissimilar in Chiroptera (mean = −0.64, SD = 0.09). Observed species pools were most similar for Lagomorpha (mean = 0.43, SD = 0.20) and were most dissimilar for Mammalia (mean = 0.91, SD = 0.03). Summary statistics for all analyzed taxa are available in Table 1.

**TABLE 1:** Summary statistics of observed data by taxa for each community metric.

Taxon	Grid cells sampled	*N* species detected (structured)	*N* species detected (unstructured)	*N* observations (structured)	*N* observations (iNaturalist)	Proportional richness difference	Jaccard dissimilarity
Mammalia	919	6.57 (3.90)	6.79 (5.72)	605.43 (2174.00)	73 (252)	−0.20 (0.23)	0.91 (0.03)
Artiodactyla	185	1.61 (0.96)	1.58 (1.01)	544.71 (867.01)	30.56 (67.12)	−0.16 (0.17)	0.37 (0.19)
Carnivora	191	4.41 (2.03)	3.35 (2.03)	370.80 (940.82)	26.57 (51.82)	−0.34 (0.19)	0.67 (0.08)
Chiroptera	123	5.50 (2.52)	1.45 (0.97)	119.52 (304.01)	2.43 (3.69)	−0.64 (0.09)	0.86 (0.04)
Lagomorpha	99	1.18 (0.56)	1.25 (0.58)	77.17 (171.17)	30.03 (66.17)	0.04 (0.12)	0.43 (0.20)
Rodentia	177	2.51 (1.39)	3.81 (2.26)	634.16 (1085.80)	98.17 (271.51)	−0.01 (0.21)	0.62 (0.10)

*Note:* Mean values per 25 × 25‐km grid cell are reported, with standard deviations listed in parentheses.

### 3.2 Species Richness

Intercept terms for Chiroptera, Carnivora, and Mammalia were significantly negative, indicating that structured surveys report greater species richness than unstructured surveys with all other variables are held at their mean. Conversely, the intercept term for Rodentia was positive, suggesting that unstructured surveys report greater rodent species richness than structured surveys with all other variables are held at their mean. Intercept terms for Artiodactyla and Lagomorpha were not significantly different from zero, suggesting that the two survey methods produce sufficiently similar estimates of species richness with all other variables are held at their mean values.

Human population density had a significant positive relationship with differences in species richness for Rodentia, Mammalia, Carnivora, Artiodactyla, and Lagomorpha. Notably, the relationships for quadratic terms were not uniformly significant, suggesting a non‐quadratic relationship between human population density and species richness for Artiodactyla and Lagomorpha. Longitude had a significant positive relationship with differences in species richness for Chiroptera and Mammalia, but Chiroptera was the only taxon significantly associated with latitude. Landscape ruggedness had a positive relationship with differences in richness for Mammalia and Artiodactyla, while the proportion of protected land had a positive relationship with differences in richness for Mammalia and Rodentia. Survey effort was only significantly associated with differences in species richness for Chiroptera. Estimates for all parameters are available in Data S3.

**FIGURE 3:**
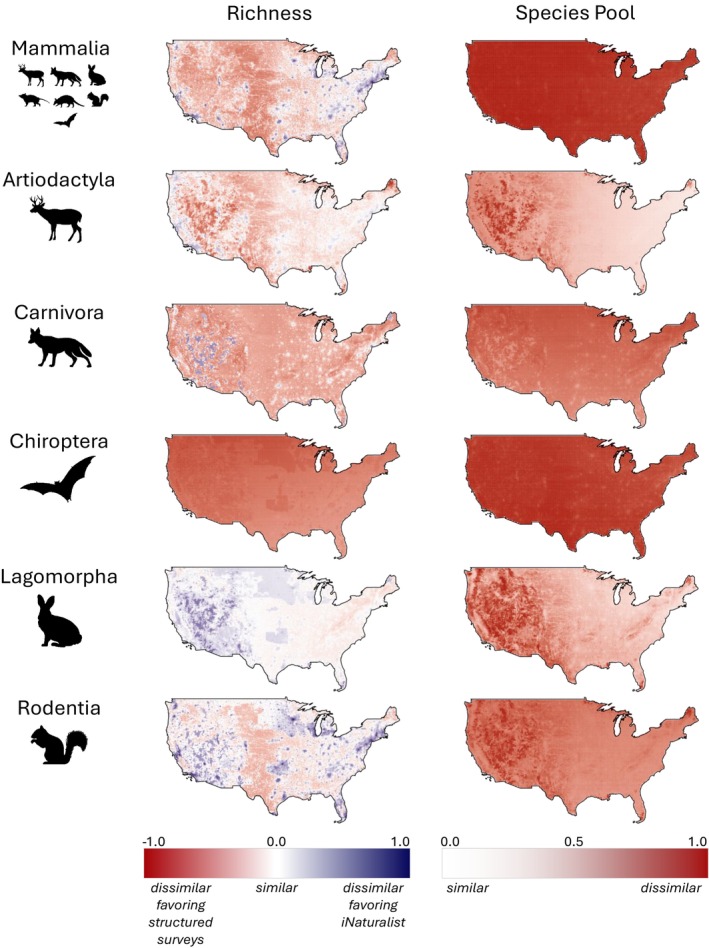
Predicted (dis)similarity of community metrics derived from iNaturalist and structured survey data across the contiguous US. Predictions are calculated holding survey effort at its mean value. Enlarged maps are available in Data S4.

Results varied substantially when estimated model coefficients were used to predict how species richness might differ between structured and unstructured surveys across the US (Figure 3). Nationally, species richness from unstructured surveys was estimated to be within 15% of richness reported from structured surveys across 87% of the contiguous US for Lagomorpha (mean = 0.04, SD = 0.12), while Rodentia (mean = −0.0004, SD = 0.21) and Artiodactyla (mean = −0.16, SD = 0.18) produced comparable datasets in 57% and 51% of grid cells, respectively. Mammalia (mean = −0.20, SD = 0.23) species richness was only comparable across 30% of grid cells, while Carnivora (mean = −0.33, SD = 0.19) was only below the 15% similarity threshold for 6% of grid cells. Species richness for Chiroptera (mean = −0.63, SD = 0.09) was not predicted to be comparable in any grid cell.

### 3.3 Species Pool

For Chiroptera, Carnivora, and Mammalia, human population density had a negative relationship with Jaccard dissimilarity—suggesting that species pools reported by both methods are likely to be more similar in areas of dense human populations. Notably, the quadratic relationship was only significant for Mammalia, while the relationship between human population density and species pool similarity was not quadratic for Carnivora and Chiroptera. Longitude had a significant negative association with species pool dissimilarity for Artiodactyla, Chiroptera, and Mammalia, while latitude had a significant positive association for Carnivora. Landscape ruggedness had a significant positive association with species pool dissimilarity for Lagomorpha, and proportion of protected land had a positive association for Mammalia. Survey effort had a negative association with species pool dissimilarity for Chiroptera and Mammalia. Intercept terms for this analysis do not have clear ecological interpretations and are thus not addressed here but are listed in Data S3.

Predicted Jaccard dissimilarity between structured and unstructured datasets was uniformly low across the continental US for most taxa. Across taxa, species pools were most similar for Artiodactyla (mean = 0.37, SD = 0.19), with approximately 8% of grid cells producing comparable datasets. Less than 1% of grid cells produced comparable species pools for Lagomorpha (mean = 0.43, SD = 0.20). Rodentia (mean = 0.62, SD = 0.10), Carnivora (mean = 0.67, SD = 0.08), Chiroptera (mean = 0.86, SD = 0.04), and Mammalia (mean = 0.91, SD = 0.03) species pools were predicted to be highly dissimilar and are not expected to produce comparable datasets in any grid cell.

## 4 Discussion

Our study found that data from structured and unstructured surveys yielded similar measures of species richness across the contiguous US but observed dissimilar species assemblages. Although relationships varied across taxa, human population density was the predominant variable associated with differences between the two datasets, followed by longitude, which we used as a proxy for local species pool breadth. Furthermore, the degree of similarity differed across taxa, indicating that the optimal survey method depends on the species being sampled. Taken together, these results suggest that iNaturalist and structured surveys may offer comparable data, but that the utility of these data depends on the taxa being sampled, the location of the survey, and the community metric being assessed. While structured surveys are typically hailed as the ‘gold standard’ of ecological research, our study suggests that data from iNaturalist can offer comparable data for some mammalian species, geographies, and community metrics.

Human population density was significantly correlated with discrepancies between structured and unstructured datasets for many taxa. Unstructured surveys typically rely on incidental encounters between human observers and non‐human organisms. Thus, unstructured surveys are expected to produce more observations in areas where more humans are available to document wildlife (Backstrom et al. 2024; Di Cecco et al. 2021; Dimson and Gillespie 2023). While densely urban landscapes support a multitude of potential wildlife observers, these same landscapes deter many species (Aronson et al. 2016). As a result, we predicted that the effect of human population density reaches a saturation point. We found evidence for a significant quadratic effect of human population density on dataset similarity in only 33% of our models. In models assessing species richness, the effect of humans was positive, which indicates that iNaturalist data from densely populated areas tend to report as many, if not more, species than structured surveys from the same areas. Furthermore, in cases where a non‐linear relationship with human population density was identified, quadratic coefficients were positive, indicating that the effect of humans increases exponentially at higher population densities rather than becoming saturated. Similarly, models assessing the similarity of species pools found a negative relationship with human population density, demonstrating that high human population densities shift metrics away from dissimilarity (*y* = 1) and toward similarity (*y* = 0). In consequence, our analysis suggests that the two survey methods produce increasingly similar datasets in areas with high human populations.

Geographic measures that coincide with species availability were similarly associated with changes in dataset similarity. Specifically, longitude tended to be significantly associated with dataset similarity, but the direction of this relationship varied. The effect of longitude was most pronounced for Chiroptera, whose datasets tended to approach similarity in the relatively species‐poor and human‐dominated eastern US (Ceballos and Ehrlich 2006; Reid 1998). Although the effect of latitude on biodiversity is well documented (Kaufman and Willig 1998), the effect of latitude was muted in comparison to the effect of longitude. We used both latitude and longitude as proxies for species richness within taxa (Kaufman and Willig 1998) and expected greater disparities between datasets in regions with larger species pools due to increased chances of collecting an incomplete sample. While this relationship held for many taxa and metrics, the variation in significance and direction of effect exemplifies the nuanced suitability of either survey method across taxa and geographies.

Contrary to our hypotheses, protected areas, landscape ruggedness, and survey effort had relatively little effect on the similarity of datasets while in the presence of the other covariates in the model. We had expected the abundance of protected areas to increase similarity between datasets since both types of surveys tend to be concentrated in protected areas as opposed to developed spaces (Carlen et al. 2024; Herrera et al. 2021). This relationship was not found to be widespread, however. Regardless of taxa, we expected rugged landscapes would favor structured surveys since iNaturalist observers tend to favor easily navigable landscapes (Mair and Ruete 2016). Instead, we found that landscape ruggedness was associated with greater richness from iNaturalist data compared to structured surveys for select taxa but did not find significant relationships for most taxa and metrics. Finally, the effect of survey effort was inconsistent across taxa and metrics. Our measure of survey effort should be interpreted as a control variable rather than a predictive variable, as it considered the volume and evenness of observations from both survey methods. We intended for this variable to account for instances of uneven sample sizes between survey methods. However, we instead found human population density to be a stronger predictor of dataset similarity, presumably because true effort in unstructured surveys is proportional to human population density but is unaffected by the volume of data collected by standardized surveys in the same region.

The degree to which iNaturalist data resembled structured survey data varied substantially across taxa. iNaturalist users disproportionately report large and charismatic species (Stoudt et al. 2022) due to both observer preferences and logistical difficulties that inhibit the reliable documentation of many species. For instance, small‐bodied animals can be difficult to see or take diagnostic photographs for accurate species identification (Barbato et al. 2021; Kays et al. 2022). We attempted to control for this limitation by omitting all non‐bat species too small to be reliably documented by camera traps from our analysis. This likely contributed to the high similarity in rodent species richness between the two datasets due to the lower effort required to report similar samples from an artificially depauperate species pool. However, filtering out small species did not preclude substantial differences in the species detected. Additionally, most mammal species are nocturnal and thus become largely undetectable to the average observer (Curti et al. 2024). Indeed, our analysis found that observations of bats—which are both small and nocturnal—have consistently low similarity between datasets. Bat data from iNaturalist may also contain patterns of variation and error not observed in other taxa since the iNaturalist platform can identify bat species based on either photographic or audio data, whereas other taxa are generally observed using photographic data only. Likewise, data from structured bat surveys can also be prone to error from automated species identification software, suggesting that discrepancies between structured and unstructured bat survey data can be attributed to misidentification as much as differences in survey effort or species detectability (Fritsch and Bruckner 2014; Goodwin and Gillam 2021; Solick et al. 2024). Furthermore, many non‐bat mammal species are difficult to identify using photographs alone (Barbato et al. 2021; Kays et al. 2022; McMullin and Allen 2022). This is especially true for rabbits and hares (Kays et al. 2022), which potentially explains why Lagomorpha reported greater dissimilarity in western regions where the Lagomorph species pool is larger and misidentification is more likely. Understanding these taxon‐specific biases and limitations can guide the use and utility of these data from unstructured surveys.

While structured and unstructured datasets have the capacity to produce comparable data for select taxa, notable discrepancies between datasets highlight that the suitability of either dataset depends on one's objectives. Data from structured and unstructured surveys may provide sufficiently similar insight if species richness of non‐bat mammals is the objective, but they report notably divergent species pools, which may not both be suitable for the research objective. The realization that the two survey methods document similar numbers of species but enumerate different species pools shows that neither method sufficiently captures all species present. Thus, although structured surveys have been hailed as the ‘gold standard’ in wildlife ecology, our analysis reveals that structured surveys fail to document many of the species that are documented in unstructured surveys (Foody 2011). This disparity, coupled with the notable geographic variation, demonstrates that the adequacy of either survey method depends on the research question and study area, precluding a one‐size‐fits‐all approach (Taraporevala et al. 2025).

Species conservation and management has the potential to elicit immense social and environmental benefits but requires accurate data to be carried out effectively (Costello et al. 2013; Stephenson and Stengel 2020; Weissgold 2024). Our analysis found that iNaturalist and structured surveys generally report comparable species richness values for many mammals. However, we caution against a complete reliance on either survey method since both methods document species that are missed by the other. These differences are especially apparent for taxa that are difficult to observe or identify. Such limitations can be overcome by only using datasets for taxa and geographies in which both survey methods are anticipated to produce similar data or by explicitly controlling for inherent differences in data by using an integrated modeling approach (e.g., Schank et al. 2017; Goldstein et al. 2025). Although no dataset is perfect, our analysis suggests that iNaturalist can adequately supplement data from structured wildlife surveys for select taxa and geographies. The judicious use of these data can contribute to the conservation and management efforts for species that are faithfully documented in unstructured surveys, thereby allowing sampling efforts to be reallocated to taxa and geographies that require a more structured survey approach.

## Author Contributions

**Daniel J. Herrera:** conceptualization (lead), data curation (lead), formal analysis (lead), investigation (lead), methodology (lead), writing – original draft (lead), writing – review and editing (lead). **Christopher M. Schalk:** conceptualization (supporting), formal analysis (supporting), funding acquisition (lead), methodology (supporting), project administration (supporting), writing – review and editing (supporting). **Alex J. Jensen:** data curation (supporting), writing – review and editing (supporting). **Benjamin R. Goldstein:** data curation (supporting), writing – review and editing (supporting). **Brigit R. Rooney:** data curation (supporting), writing – review and editing (supporting). **Roland Kays:** data curation (supporting), methodology (supporting), writing – review and editing (supporting). **William J. McShea:** data curation (supporting), writing – review and editing (supporting). **Michael V. Cove:** conceptualization (supporting), data curation (supporting), formal analysis (supporting), funding acquisition (supporting), methodology (supporting), project administration (supporting), writing – review and editing (supporting).

## Conflicts of Interest

The authors declare no conflicts of interest.

## Supporting information


Data S1.

Data S2.

Data S3.

Data S4.

## Data Availability

The data used in this analysis are publicly available via iNaturalist (https://www.inaturalist.org/), Wildlife Insights (https://www.wildlifeinsights.org/), and NABat (https://www.nabatmonitoring.org/). The filtering process for iNaturalist data is described within the methods section, and the Wildlife Insights and NABat projects whose data contributed to our analysis are listed in Data S1.

## References

[ece371805-bib-0001] Altrudi, S. 2021. “Connecting to Nature Through Tech? The Case of the iNaturalist App.” Convergence: The International Journal of Research Into New Media Technologies 27: 124–141. 10.1177/1354856520933064.

[ece371805-bib-0002] Aronson, M. F. J. , C. H. Nilon , C. A. Lepczyk , et al. 2016. “Hierarchical Filters Determine Community Assembly of Urban Species Pools.” Ecology 97: 2952–2963. 10.1002/ecy.1535. 27870023

[ece371805-bib-0003] Backstrom, L. J. , C. T. Callaghan , N. P. Leseberg , C. Sanderson , R. A. Fuller , and J. E. M. Watson . 2024. “Assessing Adequacy of Citizen Science Datasets for Biodiversity Monitoring.” Ecology and Evolution 14: e10857. 10.1002/ece3.10857. 38304273 PMC10830347

[ece371805-bib-0004] Barbato, D. , A. Benocci , M. Guasconi , and G. Manganelli . 2021. “Light and Shade of Citizen Science for Less Charismatic Invertebrate Groups: Quality Assessment of iNaturalist Nonmarine Mollusc Observations in Central Italy.” Journal of Molluscan Studies 87: eyab033. 10.1093/mollus/eyab033.

[ece371805-bib-0005] Bayraktarov, E. , G. Ehmke , J. O'Connor , et al. 2019. “Do Big Unstructured Biodiversity Data Mean More Knowledge?” Frontiers in Ecology and Evolution 6: 239. 10.3389/fevo.2018.00239.

[ece371805-bib-0006] Bullion, C. N. , and C. A. Bahlai . 2024. “Data Gap or Biodiversity Gap? Evaluating Apparent Spatial Biases in Community Science Observations of Odonata in the East‐Central United States.” PeerJ 12: e18115. 10.7717/peerj.18115. 39364368 PMC11448655

[ece371805-bib-0007] Callaghan, C. T. , A. G. B. Poore , M. Hofmann , C. J. Roberts , and H. M. Pereira . 2021. “Large‐Bodied Birds Are Over‐Represented in Unstructured Citizen Science Data.” Scientific Reports 11: 19073. 10.1038/s41598-021-98584-7. 34561517 PMC8463711

[ece371805-bib-0008] Carlen, E. J. , C. O. Estien , T. Caspi , et al. 2024. “A Framework for Contextualizing Social‐Ecological Biases in Contributory Science Data.” People and Nature 6: 377–390. 10.1002/pan3.10592.

[ece371805-bib-0009] Carter, D. 2018. “Terrain Ruggedness and Land Cover: Improved Data for Most Research Designs.” 10.7910/DVN/WXUZBN.

[ece371805-bib-0010] Ceballos, G. , and P. R. Ehrlich . 2006. “Global Mammal Distributions, Biodiversity Hotspots, and Conservation.” Proceedings. National Academy of Sciences. United States of America 103: 19374–19379. 10.1073/pnas.0609334103. PMC1698439 17164331

[ece371805-bib-0011] Chesshire, P. R. , E. E. Fischer , N. J. Dowdy , et al. 2023. “Completeness Analysis for Over 3000 United States Bee Species Identifies Persistent Data Gap.” Ecography 2023: e06584. 10.1111/ecog.06584.

[ece371805-bib-0012] Costello, M. J. , W. K. Michener , M. Gahegan , Z.‐Q. Zhang , and P. E. Bourne . 2013. “Biodiversity Data Should Be Published, Cited, and Peer Reviewed.” Trends in Ecology and Evolution 28: 454–461. 10.1016/j.tree.2013.05.002. 23756105

[ece371805-bib-0013] Curti, J. N. , M. Barton , R. G. Flores , et al. 2024. “Using Unstructured Crowd‐Sourced Data to Evaluate Urban Tolerance of Terrestrial Native Animal Species Within a California Mega‐City.” PLoS One 19: e0295476. 10.1371/journal.pone.0295476. 38809860 PMC11135677

[ece371805-bib-0014] Daru, B. H. , and J. Rodriguez . 2023. “Mass Production of Unvouchered Records Fails to Represent Global Biodiversity Patterns.” Nature Ecology and Evolution 7: 816–831. 10.1038/s41559-023-02047-3. 37127769

[ece371805-bib-0015] de Valpine, P. , D. Turek , C. J. Paciorek , C. Anderson‐Bergman , D. Temple Lang , and R. Bodik . 2017. “Programming With Models: Writing Statistical Algorithms for General Model Structures With NIMBLE.” Journal of Computational and Graphical Statistics 26: 403–413. 10.1080/10618600.2016.1172487.

[ece371805-bib-0016] Di Cecco, G. J. , V. Barve , M. W. Belitz , B. J. Stucky , R. P. Guralnick , and A. H. Hurlbert . 2021. “Observing the Observers: How Participants Contribute Data to iNaturalist and Implications for Biodiversity Science.” Bioscience 71: 1179–1188. 10.1093/biosci/biab093.

[ece371805-bib-0017] Dimson, M. , and T. W. Gillespie . 2023. “Who, Where, When: Observer Behavior Influences Spatial and Temporal Patterns of iNaturalist Participation.” Applied Geography 153: 102916. 10.1016/j.apgeog.2023.102916.

[ece371805-bib-0018] Dobson, A. D. M. , E. J. Milner‐Gulland , N. J. Aebischer , et al. 2020. “Making Messy Data Work for Conservation.” One Earth 2: 455–465. 10.1016/j.oneear.2020.04.012.

[ece371805-bib-0019] Estien, C. , E. Carlen , and C. Schell . 2024. “Examining the Influence of Sociodemographics, Residential Segregation, and Historical Redlining on eBird and iNaturalist Data Disparities in Three U.S. Cities.” Ecology and Society 29: art16. 10.5751/ES-15263-290316.

[ece371805-bib-0020] Foody, G. M. 2011. “Impacts of Imperfect Reference Data on the Apparent Accuracy of Species Presence‐Absence Models and Their Predictions: Imperfect Reference Data.” Global Ecology and Biogeography 20: 498–508. 10.1111/j.1466-8238.2010.00605.x.

[ece371805-bib-0021] Forti, L. R. , J. L. C. Da Silva , E. A. Ferreira , and J. K. Szabo . 2024. “The Implications of Estimating Rarity in Brazilian Reptiles From GBIF Data Based on Contributions From Citizen Science Versus Research Institutions.” Integrative Conservation 3: 112–126. 10.1002/inc3.53.

[ece371805-bib-0022] Fritsch, G. , and A. Bruckner . 2014. “Operator Bias in Software‐Aided Bat Call Identification.” Ecology and Evolution 4: 2703–2713. 10.1002/ece3.1122. 25077021 PMC4113294

[ece371805-bib-0023] Global Biodiversity Information Facility . 2024. “Resources Query: Peer‐Reviewed Literature [WWW Document].” https://www.gbif.org/resource/search?contentType=literature&peerReview=true.

[ece371805-bib-0024] Goldstein, B. R. , K. Pacifici , A. J. Jensen , et al. 2025. “Mammal Niches Are Not Conserved Over Continental Scales.” 10.1101/2025.01.17.633640.

[ece371805-bib-0025] Goldstein, B. R. , and S. Stoudt . 2025. “Evidence of Novelty and Specialization Behavior in Participatory Science Reporting.” Oikos 2025: e10938. 10.1111/oik.10938.

[ece371805-bib-0026] Goldstein, B. R. , S. Stoudt , J. M. Lewthwaite , V. Shirey , E. Mendoza , and L. M. Guzman . 2024. “Logistical and Preference Bias in Participatory Science Butterfly Data.” Frontiers in Ecology and the Environment 22: e2783. 10.1002/fee.2783.

[ece371805-bib-0027] Goodwin, K. R. , and E. H. Gillam . 2021. “Testing Accuracy and Agreement Among Multiple Versions of Automated Bat Call Classification Software.” Wildlife Society Bulletin 45: 690–705. 10.1002/wsb.1235.

[ece371805-bib-0028] Herrera, D. J. , S. M. Moore , D. T. T. Flockhart , W. J. McShea , and M. V. Cove . 2021. “Thinking Outside the Park: Recommendations for Camera Trapping Mammal Communities in the Urban Matrix.” Journal of Urban Ecology 7: juaa036. 10.1093/jue/juaa036.

[ece371805-bib-0029] Jacobs, C. , and A. Zipf . 2017. “Completeness of Citizen Science Biodiversity Data From a Volunteered Geographic Information Perspective.” Geo‐Spatial Information Science 20: 3–13. 10.1080/10095020.2017.1288424.

[ece371805-bib-0030] Jessup, D. A. 2003. “Opportunistic Research and Sampling Combined With Fish and Wildlife Action Management Actions or Crisis Response.” Institute for Laboratory Animal Research Journal 44: 277–285. 10.1093/ilar.44.4.277. 13130158

[ece371805-bib-0031] Kaufman, D. M. , and M. R. Willig . 1998. “Latitudinal Patterns of Mammalian Species Richness in the New World: The Effects of Sampling Method and Faunal Group.” Journal of Biogeography 25: 795–805. 10.1046/j.1365-2699.1998.2540795.x.

[ece371805-bib-0032] Kays, R. , M. Lasky , M. L. Allen , et al. 2022. “Which Mammals Can Be Identified From Camera Traps and Crowdsourced Photographs?” Journal of Mammalogy 103: 767–775. 10.1093/jmammal/gyac021.

[ece371805-bib-0033] Kindsvater, H. K. , N. K. Dulvy , C. Horswill , M.‐J. Juan‐Jordá , M. Mangel , and J. Matthiopoulos . 2018. “Overcoming the Data Crisis in Biodiversity Conservation.” Trends in Ecology and Evolution 33: 676–688. 10.1016/j.tree.2018.06.004. 30007845

[ece371805-bib-0034] Li, W. , Q. Guo , and C. Elkan . 2011. “Can We Model the Probability of Presence of Species Without Absence Data?” Ecography 34: 1096–1105.

[ece371805-bib-0035] Loeb, S. C. , T. J. Rodhouse , L. E. Ellison , et al. 2015. “A Plan for the North American Bat Monitoring Program (NABat).”

[ece371805-bib-0036] López‐Guillén, E. , I. Herrera , B. Bensid , et al. 2024. “Strengths and Challenges of Using iNaturalist in Plant Research With Focus on Data Quality.” Diversity 16: 42. 10.3390/d16010042.

[ece371805-bib-0037] MacKenzie, D. I. 2005a. “What Are the Issues With Presence‐Absence Data for Wildlife Managers?” Journal of Wildlife Management 69: 849–860.

[ece371805-bib-0038] Mackenzie, D. I. 2005b. “Was It There? Dealing With Imperfect Detection for Species Presence/Absence Data.” Australian and New Zealand Journal of Statistics 47: 65–74. 10.1111/j.1467-842X.2005.00372.x.

[ece371805-bib-0039] Mair, L. , and A. Ruete . 2016. “Explaining Spatial Variation in the Recording Effort of Citizen Science Data Across Multiple Taxa.” PLoS One 11: e0147796. 10.1371/journal.pone.0147796. 26820846 PMC4731209

[ece371805-bib-0040] Martínez‐Sagarra, G. , F. Castilla , and F. Pando . 2022. “Seven Hundred Projects in iNaturalist Spain: Performance and Lessons Learned.” Sustainability 14: 11093. 10.3390/su141711093.

[ece371805-bib-0041] McMullin, R. T. , and J. L. Allen . 2022. “An Assessment of Data Accuracy and Best Practice Recommendations for Observations of Lichens and Other Taxonomically Difficult Taxa on iNaturalist.” Botany 100: 491–497. 10.1139/cjb-2021-0160.

[ece371805-bib-0042] Mesaglio, T. , and C. T. Callaghan . 2021. “An Overview of the History, Current Contributions and Future Outlook of iNaturalist in Australia.” Wildlife Research 48: 289–303. 10.1071/WR20154.

[ece371805-bib-0043] Morin, D. J. , S. D. Higdon , J. L. Holub , et al. 2016. “Bias in Carnivore Diet Analysis Resulting From Misclassification of Predator Scats Based on Field Identification.” Wildlife Society Bulletin 40: 669–677. 10.1002/wsb.723.

[ece371805-bib-0044] NASA Socioeconomic Data and Applications Center . 2018. “Gridded Population of the World, Version 4 (GPWv4): Population Count, Revision 11.” 10.7927/H4JW8BX5.

[ece371805-bib-0045] Oksanen, J. , G. L. Simpson , F. G. Blanchet , et al. 2022. “Vegan: Community Ecology Package.”

[ece371805-bib-0046] Oliver, P. M. , A. Davie‐Rieck , M. I. Ramdani , et al. 2025. “Can Citizen Science Fill Knowledge Gaps for the World's Most Speciose and Poorly‐Known Insular Amphibian Fauna?” Pacific Conservation Biology 31: PC24063. 10.1071/PC24063.

[ece371805-bib-0047] Périquet, S. , L. Roxburgh , A. Le Roux , and W. J. Collinson . 2018. “Testing the Value of Citizen Science for Roadkill Studies: A Case Study From South Africa.” Frontiers in Ecology and Evolution 6: 15. 10.3389/fevo.2018.00015.

[ece371805-bib-0048] Phillips, S. J. , R. P. Anderson , and R. E. Schapire . 2006. “Maximum Entropy Modeling of Species Geographic Distributions.” Ecological Modelling 190: 231–259. 10.1016/j.ecolmodel.2005.03.026.

[ece371805-bib-0049] Reid, W. V. 1998. “Biodiversity Hotspots.” Trends in Ecology and Evolution 13: 275–280. 10.1016/S0169-5347(98)01363-9. 21238297

[ece371805-bib-0050] Rooney, B. , R. Kays , M. V. Cove , et al. 2025. “SNAPSHOT USA 2019–2023: The First Five Years of Data From a Coordinated Camera Trap Survey of the United States.” Global Ecology and Biogeography 34: e13941. 10.1111/geb.13941.

[ece371805-bib-0051] Schank, C. J. , M. V. Cove , M. J. Kelly , et al. 2017. “Using a Novel Model Approach to Assess the Distribution and Conservation Status of the Endangered Baird's Tapir.” Diversity and Distributions 23: 1459–1471. 10.1111/ddi.12631.

[ece371805-bib-0052] Shamon, H. , R. Maor , M. V. Cove , et al. 2024. “SNAPSHOT USA 2021: A Third Coordinated National Camera Trap Survey of the United States.” Ecology 105: e4318. 10.1002/ecy.4318. 38693703

[ece371805-bib-0053] Shirey, V. , M. W. Belitz , V. Barve , and R. Guralnick . 2021. “A Complete Inventory of North American Butterfly Occurrence Data: Narrowing Data Gaps, but Increasing Bias.” Ecography 44: 537–547. 10.1111/ecog.05396.

[ece371805-bib-0054] Solick, D. I. , B. H. Hopp , J. Chenger , and C. M. Newman . 2024. “Automated Echolocation Classifiers Vary in Accuracy for Northeastern U.S. Bat Species.” PLoS One 19: e0300664. 10.1371/journal.pone.0300664. 38829847 PMC11146688

[ece371805-bib-0055] Spitzer, R. , M. Churski , A. Felton , et al. 2019. “Doubting Dung: eDNA Reveals High Rates of Misidentification in Diverse European Ungulate Communities.” European Journal of Wildlife Research 65: 28. 10.1007/s10344-019-1264-8.

[ece371805-bib-0056] Stephenson, P. J. , and C. Stengel . 2020. “An Inventory of Biodiversity Data Sources for Conservation Monitoring.” PLoS One 15: e0242923. 10.1371/journal.pone.0242923. 33264320 PMC7710106

[ece371805-bib-0057] Stoudt, S. , B. R. Goldstein , and P. De Valpine . 2022. “Identifying Engaging Bird Species and Traits With Community Science Observations.” Proceedings of the National Academy of Sciences of the United States of America 119: e2110156119. 10.1073/pnas.2110156119. 35412904 PMC9169790

[ece371805-bib-0058] Taraporevala, N. F. , J. P. Beckmann , and J. K. Young . 2025. “Citizen Science Project on Urban Canids Provides Different Results From Camera Traps but Generates Interest and Revenue.” Wildlife Biology: e01382. 10.1002/wlb3.01382.

[ece371805-bib-0059] Troudet, J. , R. Vignes‐Lebbe , P. Grandcolas , and F. Legendre . 2018. “The Increasing Disconnection of Primary Biodiversity Data From Specimens: How Does It Happen and How to Handle It?” Systematic Biology 67: 1110–1119. 10.1093/sysbio/syy044. 29893962

[ece371805-bib-0060] United States Geological Survey . 2024. “PAD‐US.”

[ece371805-bib-0061] Viola, B. , P. Puskic , S. Corney , et al. 2024. “A Quantitative Assessment of Continuous Versus Structured Methods for the Detection of Marine Mammals and Seabirds via Opportunistic Shipboard Surveys.” Scientific Reports 14: 18796. 10.1038/s41598-024-68512-6. 39138319 PMC11322172

[ece371805-bib-0062] Wahlberg, A. C. , R. Antoniazzi , and C. M. Schalk . 2023. “Patterns of the Introduction, Spread, and Impact of the Brown Widow Spider, *Latrodectus geometricus* (Araneae: Theridiidae), in the Americas.” Journal of Arachnology 51: 195–205. 10.1636/JoA-S-22-022.

[ece371805-bib-0063] Weissgold, B. J. 2024. “US Wildlife Trade Data Lack Quality Control Necessary for Accurate Scientific Interpretation and Policy Application.” Conservation Letters 17: e13005. 10.1111/conl.13005.

[ece371805-bib-0064] Wilman, H. , J. Belmaker , J. Simpson , C. de la Rosa , M. M. Rivadeneira , and W. Jetz . 2014. “EltonTraits 1.0: Species‐Level Foraging Attributes of the World's Birds and Mammals.” Ecology 95: 2027. 10.1890/13-1917.1.

