# Supplementary material for: iNaturalist and Structured Mammal Surveys Reflect Similar Species Richness but Capture Different Species Pools Across the United States
Source: Ecol Evol. 2025 Jul 20;15(7):e71805. doi: 10.1002/ece3.71805 (PMC12276820; doi:10.1002/ece3.71805)
Supplement: Supplementary file 1 — Data S1. [file ECE3-15-e71805-s001.docx]

**Supplemental Table 1.1**. Auxiliary data sources from public camera trapping projects hosted on Wildlife Insights. Detection data from all listed surveys were used in this analysis.

| **Project name** | **Sample years used** | **Citation** |
| --- | --- | --- |
| Cascades Wolverine Project | 2021 | Williams, Stephanie; Moskowitz, David E. 2017. Last updated March 2025. Cascades Wolverine Project. http://n2t.net/ark:/63614/w12001678. Accessed via wildlifeinsights.org on 2024-07-06. |
| Cibola Monitoring | 2021-2022 | Talken, A. 2022. Last updated June 2023. Cibola Monitoring. http://n2t.net/ark:/63614/w12006101. Accessed via wildlifeinsights.org on 2024-07-06. |
| Humboldt Marten Distribution and Density | 2021 | Martin, Marie E, Green, David S, and Matthews, Sean M. 2021. Last updated February 2025. Humboldt Marten Distribution and Density. http://n2t.net/ark:/63614/w12003402. Accessed via wildlifeinsights.org on 2024-07-06. |
| Klamath Carnivore Community Project | 2021 | Martin, Marie E., Green, David S., and Matthews, Sean M. 2018. Last updated March 2025. Klamath Carnivore Community Project. http://n2t.net/ark:/63614/w12001294. Accessed via wildlifeinsights.org on 2024-07-06. |
| Temecula Creek Bridge | 2021-2022 | Vu, L., vickers, w., Gonzalez, J. 2020. Last updated August 2022. Temecula Creek Bridge. http://n2t.net/ark:/63614/w12003507. Accessed via wildlifeinsights.org on 2024-07-06. |
| Wallowa Wolverine & Forest Carnivore Project | 2021-2022 | Dreher, Kayla A. Shively, Scott C. Magoun, Audrey J. 2019. Last updated January 2024. Wallowa Wolverine & Forest Carnivore Project. http://n2t.net/ark:/63614/w12003809. Accessed via wildlifeinsights.org on 2024-07-06. |

**Supplemental Table 1.2**. Data sources from bat surveys hosted on NABat. Acoustic data from all listed surveys were used in this analysis.

| **Project name** | **Sample years used** | **Recording device** | **Acoustic identification software** |
| --- | --- | --- | --- |
| USAF East Region | 2021, 2022 | WILDLIFE ACOUSTICS SM4BAT-FS, PETTERSSON D500x, | Wildlife Acoustics Kaleidoscope 5.1.x, Sonobat 4.x |
| Naval Base Ventura County Bats | 2021, 2022 | WILDLIFE ACOUSTICS SM3Bat, WILDLIFE ACOUSTICS SM4BAT-FS | Sonobat 4.x |
| Arnold Air Force Base NABat Monitoring | 2021, 2022 | Not listed | Not listed |
| North Fork Hughes River | 2021 | WILDLIFE ACOUSTICS SM4BAT-ZC | Wildlife Acoustics Kaleidoscope 5.4.x |
| EFMO Bat Monitoring | 2021, 2022 | WILDLIFE ACOUSTICS SM4BAT-FS, WILDLIFE ACOUSTICS SM3Bat | Wildlife Acoustics Kaleidoscope x, Wildlife Acoustics Kaleidoscope 5.4.x |
| San Luis National Wildlife Refuge Complex Bat Survey | 2021 | PETTERSSON D500x | Sonobat 4.x |
| Year-round Monitoring Pacific Northwest | 2021 | PETTERSSON D500x, WILDLIFE ACOUSTICS SM4BAT-FS | Sonobat 4.2 |
| Spirit of the Wild Metadata | 2021, 2022 | WILDLIFE ACOUSTICS SM4BAT-FS | Wildlife Acoustics Kaleidoscope 5.1.x, Wildlife Acoustics Kaleidoscope 5.2.x, Wildlife Acoustics Kaleidoscope 5.4.x |
| NPS Mojave Desert Network Bat Monitoring | 2021, 2022 | PETTERSSON D500x | Sonobat 4.x |
| Hungry Valley SVRA Bat Program | 2021, 2022 | WILDLIFE ACOUSTICS SM4BAT-FS | Sonobat 4.x |
| CAVENPS | 2021 | WILDLIFE ACOUSTICS SM4BAT-FS | Wildlife Acoustics Kaleidoscope 5.3.x, Sonobat 4.x |
| Upper Mississippi River National Wildlife and Fish Refuge Bat Survey | 2021, 2022 | WILDLIFE ACOUSTICS SM4BAT-FS | Wildlife Acoustics Kaleidoscope 5.2.x |
| Summit Lake Paiute Tribe Bat Inventory and Monitoring Project | 2021, 2022 | WILDLIFE ACOUSTICS SM4BAT-FS, | Sonobat 4.x |
| 2020-2021 HCSF Spring Emergence and Fall Swarming | 2021 | TITLEY AnaBat SD2 | Wildlife Acoustics Kaleidoscope 5.4.x |
| USFWS Inland SoCal Monitoring | 2021 | TITLEY AnaBat Swift | Sonobat 4.x |
| Acadia National Park Active Period Monitoring | 2021 | PETTERSSON D500x | Sonobat 4.x |
| Somerset Armory Planning Level Survey 2021 | 2021 | WILDLIFE ACOUSTICS SMZC, WILDLIFE ACOUSTICS SM3Bat | Wildlife Acoustics Kaleidoscope x |
| AZGFD NABat Project | 2021, 2022 | WILDLIFE ACOUSTICS SMMINI-BAT, WILDLIFE ACOUSTICS SM4BAT-FS, WILDLIFE ACOUSTICS SM4BAT | Sonobat 4.x |
| Acadia National Park Randomized Grid Cell Monitoring | 2021, 2022 | PETTERSSON D500x | Sonobat 4.x |
| Forest Service Region 3 | 2021, 2022 | WILDLIFE ACOUSTICS SM4BAT-FS, WILDLIFE ACOUSTICS SM3Bat, WILDLIFE ACOUSTICS SM4BAT | Sonobat 4.x, Sonobat 30 |
| NDOW NABat in Nevada | 2021, 2022 | TITLEY AnaBat Swift, , PETTERSSON D500x, WILDLIFE ACOUSTICS SMMINI-BAT | Sonobat 4.x |
| BLM California Desert Conservation Area | 2021, 2022 | TITLEY AnaBat Swift, WILDLIFE ACOUSTICS SM3Bat, TITLEY AnaBat Express | Sonobat 4.x |
| Bureau of Land Management-Royal Gorge Field Office Bat Monitoring | 2021, 2022 | WILDLIFE ACOUSTICS SM4BAT-FS | Sonobat 4.2, Sonobat 4.x |
| Forest Service Region 5 | 2021, 2022 | WILDLIFE ACOUSTICS SM4BAT-FS, WILDLIFE ACOUSTICS SM3Bat, WILDLIFE ACOUSTICS SMMINI-BAT, WILDLIFE ACOUSTICS SM4BAT | Sonobat 4.x, Sonobat 30 |
| USFS Region 5 | 2021, 2022 | WILDLIFE ACOUSTICS SM4BAT-FS, WILDLIFE ACOUSTICS SM3Bat, WILDLIFE ACOUSTICS SMMINI-BAT, WILDLIFE ACOUSTICS SM4BAT | Sonobat 4.x, Sonobat 30 |
| McDowell Sonoran Preserve Bat Acoustics | 2021, 2022 | WILDLIFE ACOUSTICS SMMINI-BAT | Sonobat 4.x |
| Forest Service Region 8 | 2021 | WILDLIFE ACOUSTICS SMMINI-BAT | Sonobat 4.x |
| Bishop BLM | 2021, 2022 | WILDLIFE ACOUSTICS SM4BAT-FS | Sonobat 4.x |
| Apalachicola National Forest | 2021, 2022 | WILDLIFE ACOUSTICS SM4BAT | Wildlife Acoustics Kaleidoscope 5.4.x |
| Ohio River Islands NWR | 2021, 2022 | PETTERSSON D500x, | Sonobat 4.x |
| Hillsborough County Bat Monitoring | 2021 | WILDLIFE ACOUSTICS SM4BAT, WILDLIFE ACOUSTICS SM3Bat | Wildlife Acoustics Kaleidoscope 5.3.x |
| USGS-FORT NABat Monitoring | 2021, 2022 | WILDLIFE ACOUSTICS SM4BAT-FS, WILDLIFE ACOUSTICS SM3Bat, AudioMoth 1.2.0 | Wildlife Acoustics Kaleidoscope 5.3.x, Sonobat 4.x, Wildlife Acoustics Kaleidoscope x, Wildlife Acoustics Kaleidoscope 5.4.x |
| BLM Carslbad Field Office New Mexico | 2021, 2022 | WILDLIFE ACOUSTICS SM4BAT-FS | Sonobat 4.x |
| BLM Uncompahgre Field Office (UFO) Acoustic Monitoring | 2021, 2022 | WILDLIFE ACOUSTICS SM4BAT-FS | Sonobat 4.4.5, Sonobat 4.x |
| Colorado NABat Monitoring | 2021, 2022 | WILDLIFE ACOUSTICS SM4BAT-FS, WILDLIFE ACOUSTICS SM2Bat+, WILDLIFE ACOUSTICS SM3Bat, | Sonobat 4.2, Sonobat 4.4.5 |
| Wisconsin DNR Acoustic Monitoring | 2021, 2022 | TITLEY AnaBat SD2, TITLEY AnaBat Swift, | Wildlife Acoustics Kaleidoscope x |
| South Carolina NABat acoustic | 2021, 2022 | TITLEY AnaBat SD2, TITLEY AnaBat Swift | Wildlife Acoustics Kaleidoscope 5.4.x, Wildlife Acoustics Kaleidoscope x |
| Duke Energy: North Carolina and South Carolina | 2021 | WILDLIFE ACOUSTICS SM4BAT-FS, WILDLIFE ACOUSTICS SM4BAT | Wildlife Acoustics Kaleidoscope 5.4.x |
| NABat North Carolina stationary survey | 2021, 2022 | TITLEY AnaBat SD2, | Wildlife Acoustics Kaleidoscope 4.0.0, |
| BLM Gunnison Field Office NABat Monitoring | 2021 | WILDLIFE ACOUSTICS SM4BAT-FS | Sonobat 4.2 |
| Elko BLM Biodiversity Program | 2021, 2022 | WILDLIFE ACOUSTICS SM4BAT-FS | Sonobat 4.x |
| BLM NV WDO NABat Monitoring | 2021 | PETTERSSON D500x | Sonobat 4.x |
| Yurok Tribe Bat Inventory and Monitoring Project | 2021, 2022 | WILDLIFE ACOUSTICS SMMINI-BAT | Sonobat 4.x |
| NW NABat | 2021, 2022 | PETTERSSON D500x, WILDLIFE ACOUSTICS SM4BAT-FS, TITLEY AnaBat Swift, WILDLIFE ACOUSTICS SM2Bat+ | Sonobat 4.2 |
| National Wildlife Refuges Pacific Northwest Region 1 (R1) | 2021, 2022 | PETTERSSON D500x, PETTERSSON D1000x, WILDLIFE ACOUSTICS SM4BAT-FS, PETTERSSON D240x | Sonobat 4.x, Sonobat 4.2 |
| Fort Leavenworth Bat surveys | 2021, 2022 | WILDLIFE ACOUSTICS SMMINI-BAT | Wildlife Acoustics Kaleidoscope 5.4.x |
| NPS Klamath region NABat cells | 2021, 2022 | PETTERSSON D500x, PETTERSSON D1000x, WILDLIFE ACOUSTICS SMMINI-BAT | Sonobat 4.x |
| SoCal Bat Working Group Volunteer Monitoring | 2021, 2022 | PETTERSSON D500x, WILDLIFE ACOUSTICS SM3Bat, WILDLIFE ACOUSTICS SMMINI-BAT | Sonobat 4.x |
| USACE Lake Wappapello | 2021, 2022 | WILDLIFE ACOUSTICS SM4BAT-FS | Wildlife Acoustics Kaleidoscope 5.4.x |
| Marin Bat Monitoring | 2021, 2022 | PETTERSSON D500x | Sonobat 4.x |
| NABat data collection in Curecanti NRA and Black Canyon of the Gunnison NP | 2021, 2022 | WILDLIFE ACOUSTICS SM2Bat+, WILDLIFE ACOUSTICS SM4BAT-FS | Sonobat 4.x |
| NM Mining and Minerals Division | 2021 | WILDLIFE ACOUSTICS SM4BAT-FS, WILDLIFE ACOUSTICS SM3Bat | Sonobat 4.x |
| Navajo Nation Bat Program | 2021, 2022 | WILDLIFE ACOUSTICS SMMINI-BAT, AudioMoth 1.2.0 | Sonobat 4.x |
| Forest Service Region 4 NABat-NW Bat Hub | 2021, 2022 | PETTERSSON D500x, WILDLIFE ACOUSTICS SM4BAT-FS, AudioMoth 1.2.0 | Sonobat 4.x |
| Morgan County IN | 2021 | TITLEY AnaBat Swift | Wildlife Acoustics Kaleidoscope 5.4.x |
| MNDNR Acoustic Routes | 2021, 2022 | WILDLIFE ACOUSTICS SM4BAT-FS, WILDLIFE ACOUSTICS SM3Bat | Sonobat 4.x, Wildlife Acoustics Kaleidoscope 5.4.x |
| Forest Service Region 9 | 2021, 2022 | WILDLIFE ACOUSTICS SM4BAT-FS, WILDLIFE ACOUSTICS SM3Bat, PETTERSSON D500x, TITLEY AnaBat Express | Sonobat 4.x, Wildlife Acoustics Kaleidoscope 5.3.x |
| Rocky Mountain National Park NABat | 2021 | WILDLIFE ACOUSTICS SM4BAT-FS, WILDLIFE ACOUSTICS SMMINI-BAT, PETTERSSON D500x | Sonobat 4.2 |
| Oregon Caves Bat Acoustics | 2021, 2022 | PETTERSSON D500x | Sonobat 4.x, Sonobat 4.2 |
| Bandelier and Valles Caldera | 2021, 2022 | WILDLIFE ACOUSTICS SM4BAT-FS, WILDLIFE ACOUSTICS SMMINI-BAT | Sonobat 4.x |
| Camp Rilea | 2021, 2022 | PETTERSSON D500x | Sonobat 4.x |
| Pyramid Lake Paiute Tribe | 2021, 2022 | WILDLIFE ACOUSTICS SM4BAT-FS, WILDLIFE ACOUSTICS SM4BAT | Sonobat 4.x, Wildlife Acoustics Kaleidoscope 5.4.x |
| Fish Eating Creek Metadata | 2021, 2022 | WILDLIFE ACOUSTICS SM4BAT-FS | Wildlife Acoustics Kaleidoscope 5.4.x |
| Forest Service - Ozark-St. Francis National Forests | 2021, 2022 | WILDLIFE ACOUSTICS SMMINI-BAT | Wildlife Acoustics Kaleidoscope 5.2.x, Sonobat 4.x |
| Dinner Island Ranch MetaData | 2021, 2022 | WILDLIFE ACOUSTICS SM4BAT-FS | Wildlife Acoustics Kaleidoscope 5.4.x |
| Iowa Acoustic Bat Monitoring | 2021, 2022 | WILDLIFE ACOUSTICS SM4BAT-FS | Wildlife Acoustics Kaleidoscope x, Wildlife Acoustics Kaleidoscope 5.4.x |
| Camp Blanding Joint Training Center | 2021, 2022 | WILDLIFE ACOUSTICS SM4BAT-FS | Wildlife Acoustics Kaleidoscope 5.1.x, Wildlife Acoustics Kaleidoscope 5.4.x |
| MMSF Summer Monitoring | 2021 | TITLEY AnaBat SD2 | Wildlife Acoustics Kaleidoscope 5.4.x |
| Massachusetts Department of Transportation Bat Acoustic Monitoring | 2021 | BINARY ACOUSTIC AR125 | Wildlife Acoustics Kaleidoscope 5.1.x |
| Camp Adair | 2021, 2022 | PETTERSSON D500x | Sonobat 4.x |
| BLM TRFO NABAT Monitoring | 2021, 2022 | TITLEY AnaBat Swift | Sonobat 4.x |
| Bear Knob | 2021 | WILDLIFE ACOUSTICS SM4BAT-FS | Wildlife Acoustics Kaleidoscope 5.4.x |
| Kartchner Caverns SP Pre-WNS Baseline | 2021, 2022 | TITLEY AnaBat Swift | Sonobat 4.x |
| BLM Phoenix District Office NA Bat Monitoring | 2021, 2022 | WILDLIFE ACOUSTICS SM4BAT-FS, WILDLIFE ACOUSTICS SMMINI-BAT | Sonobat 4.x |
| VTrans Acoustic Surveys | 2021 | WILDLIFE ACOUSTICS SM4BAT-FS | Wildlife Acoustics Kaleidoscope 5.1.x |
| Acadia National Park NABat Monitoring | 2021, 2022 | PETTERSSON D500x | Sonobat 4.x |
| Rhode Island Department of Transportation 2021 | 2021 | WILDLIFE ACOUSTICS SM4BAT-FS | Wildlife Acoustics Kaleidoscope 5.1.x |
| Pike National Forest and Adjacent Colorado State Wildlife Areas | 2021, 2022 | TITLEY AnaBat Swift | Sonobat 4.x |
| BLM Idaho Falls District Legacy Data | 2021 | TITLEY AnaBat Express | Wildlife Acoustics Kaleidoscope 5.3.x |
| Arkansas Acoustic Records, Pedro Ardapple | 2021, 2022 | TITLEY AnaBat Express, WILDLIFE ACOUSTICS SM4BAT-FS, WILDLIFE ACOUSTICS SM4BAT | Wildlife Acoustics Kaleidoscope 4.x, Wildlife Acoustics Kaleidoscope 5.4.x |
| Homestead National Monument of America | 2021, 2022 | WILDLIFE ACOUSTICS SM4BAT-FS | Wildlife Acoustics Kaleidoscope 5.4.x |
| Southeast Arizona Group National Park Units | 2021, 2022 | PETTERSSON D500x, PETTERSSON D1000x | Sonobat 4.x |
| Oklahoma Perimyotis Monitoring Project | 2022 | WILDLIFE ACOUSTICS SM3Bat | Wildlife Acoustics Kaleidoscope 3.x |
| Bunnells Run | 2022 | WILDLIFE ACOUSTICS SM4BAT-ZC | Wildlife Acoustics Kaleidoscope 5.4.x |
| Edgewood Open Space | 2022 | TITLEY AnaBat SD1 | Not listed |
| Florida Fish and Wildlife Conservation Commission | 2022 | WILDLIFE ACOUSTICS SM4BAT-FS | Sonobat 4.x |
| Petrified Forest Bat Monitoring | 2022 | WILDLIFE ACOUSTICS SM4BAT-FS | Sonobat 4.x |
| NABat One Health Project - Arizona | 2022 | AudioMoth 1.2.0, WILDLIFE ACOUSTICS SMMINI-BAT | Sonobat 4.x |
| US 6219 Section 050 Acoustic Presence Absence | 2022 | PETTERSSON D500x | Wildlife Acoustics Kaleidoscope 5.4.x |
| USAF West Region | 2022 | WILDLIFE ACOUSTICS SM4BAT, WILDLIFE ACOUSTICS SM4BAT-FS | Sonobat 4.x |
| BLM Gunnison Field Office (GFO) Acoustic Monitoring | 2022 | WILDLIFE ACOUSTICS SM4BAT-FS | Sonobat 4.x |
| Midwest Bat Hub Monitoring | 2022 | AudioMoth 1.0.0, TITLEY AnaBat Express, TITLEY AnaBat Swift | Wildlife Acoustics Kaleidoscope x |
| PacWest One Health | 2022 | AudioMoth 1.2.0, PETTERSSON D500x, WILDLIFE ACOUSTICS SMMINI-BAT, WILDLIFE ACOUSTICS SM MICRO | Sonobat 4.x |
| NABat One Health multiple states | 2022 | AudioMoth 1.2.0 | Sonobat 4.x |
| Stationary Acoustic Data Submission - Normandeau Associates 2022 | 2022 | BINARY ACOUSTIC iFR-V | Wildlife Acoustics Kaleidoscope 5.4.x |
| NABat One Health Project - New Mexico | 2022 | AudioMoth 1.2.0 | Sonobat 4.x |
| USFS R2 - Colorado | 2022 | WILDLIFE ACOUSTICS SM4BAT-FS | Sonobat 4.4.5 |
| Okaloacoochee Slough Metadata | 2022 | WILDLIFE ACOUSTICS SM4BAT-FS | Wildlife Acoustics Kaleidoscope 5.4.x |
| BLM NM Taos Field Office | 2022 | WILDLIFE ACOUSTICS SM4BAT-FS | Sonobat 4.x |
| USAF Midwest Region | 2022 | WILDLIFE ACOUSTICS SM4BAT-FS | Sonobat 4.x |
| Tensleep Canyon Bat Monitoring | 2022 | WILDLIFE ACOUSTICS SM4BAT-FS, WILDLIFE ACOUSTICS SM4BAT | Wildlife Acoustics Kaleidoscope 5.2.x |
| MassDOT Acoustic Rare Bat Surveys | 2022 | WILDLIFE ACOUSTICS SM4BAT-FS | Wildlife Acoustics Kaleidoscope 5.4.x |
| Eagle Lake BLM | 2022 | WILDLIFE ACOUSTICS SMMINI-BAT | Sonobat 4.x |
| Yakama Nation | 2022 | WILDLIFE ACOUSTICS SMMINI-BAT | Wildlife Acoustics Kaleidoscope 5.4.x |
| Midpeninsula Regional Open Space District | 2022 | WILDLIFE ACOUSTICS SMMINI-BAT | Sonobat 4.x |
| Forest Service Wildfire Conservation Strategy | 2022 | WILDLIFE ACOUSTICS SM4BAT-FS | Sonobat 30 |
