# Supplementary material for: iNaturalist and Structured Mammal Surveys Reflect Similar Species Richness but Capture Different Species Pools Across the United States
Source: Ecol Evol. 2025 Jul 20;15(7):e71805. doi: 10.1002/ece3.71805 (PMC12276820; doi:10.1002/ece3.71805)

**Supplemental Figure 2.1**. Model diagnostics for the Artiodactyla richness model. The posterior predictive plot (left) compares the observed density of values (blue) against the predicted density of values using the estimated model coefficients and the training dataset. Trace plots (right) show mixing of MCMC chains.
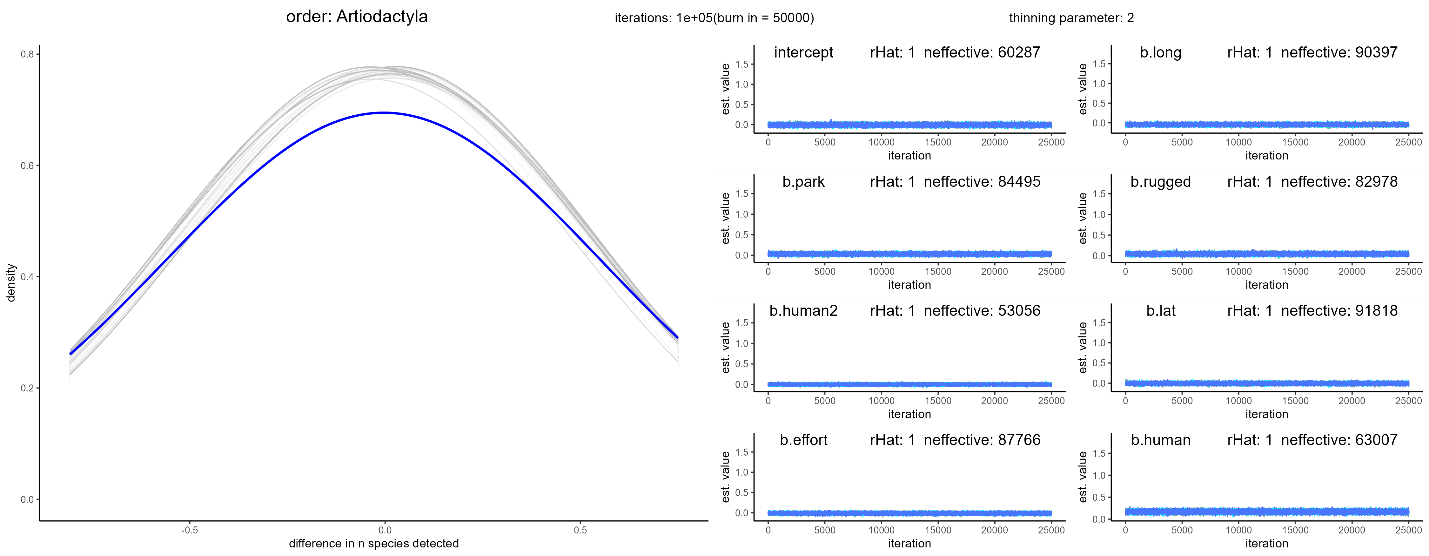


**Supplemental Figure 2.2**. Model diagnostics for the Carnivora richness model. The posterior predictive plot (left) compares the observed density of values (blue) against the predicted density of values using the estimated model coefficients and the training dataset. Trace plots (right) show mixing of MCMC chains.


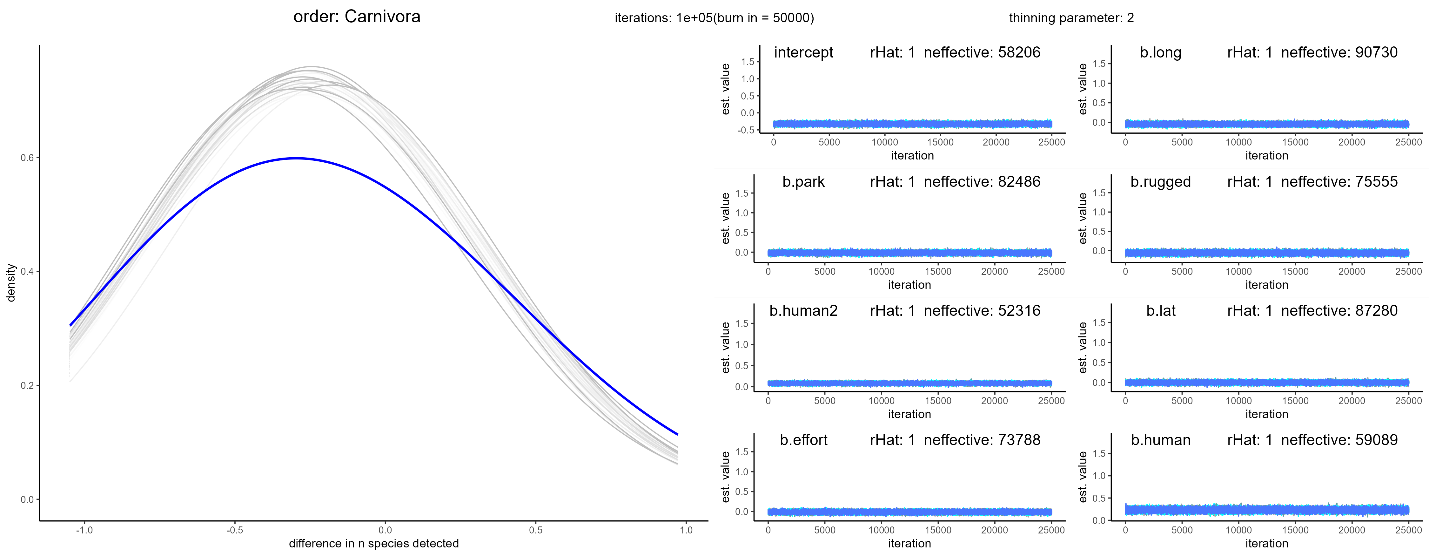


**Supplemental Figure 2.3**. Model diagnostics for the Chiroptera richness model. The posterior predictive plot (left) compares the observed density of values (blue) against the predicted density of values using the estimated model coefficients and the training dataset. Trace plots (right) show mixing of MCMC chains.
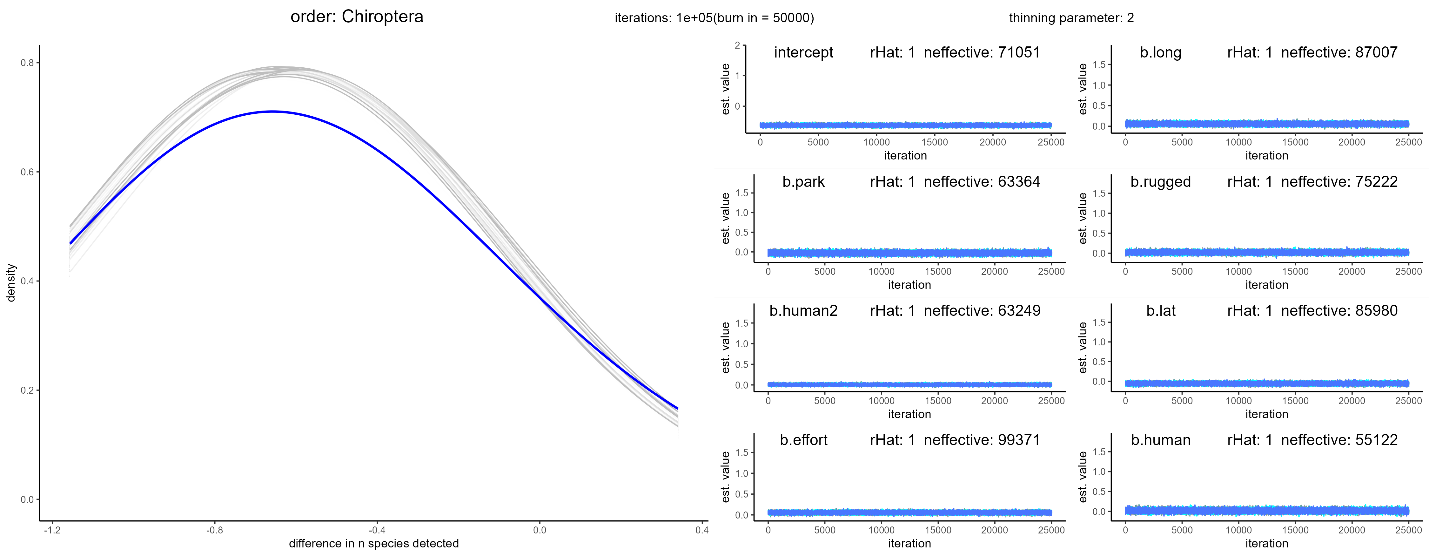


**Supplemental Figure 2.4**. Model diagnostics for the Lagomorph richness model. The posterior predictive plot (left) compares the observed density of values (blue) against the predicted density of values using the estimated model coefficients and the training dataset. Trace plots (right) show mixing of MCMC chains.
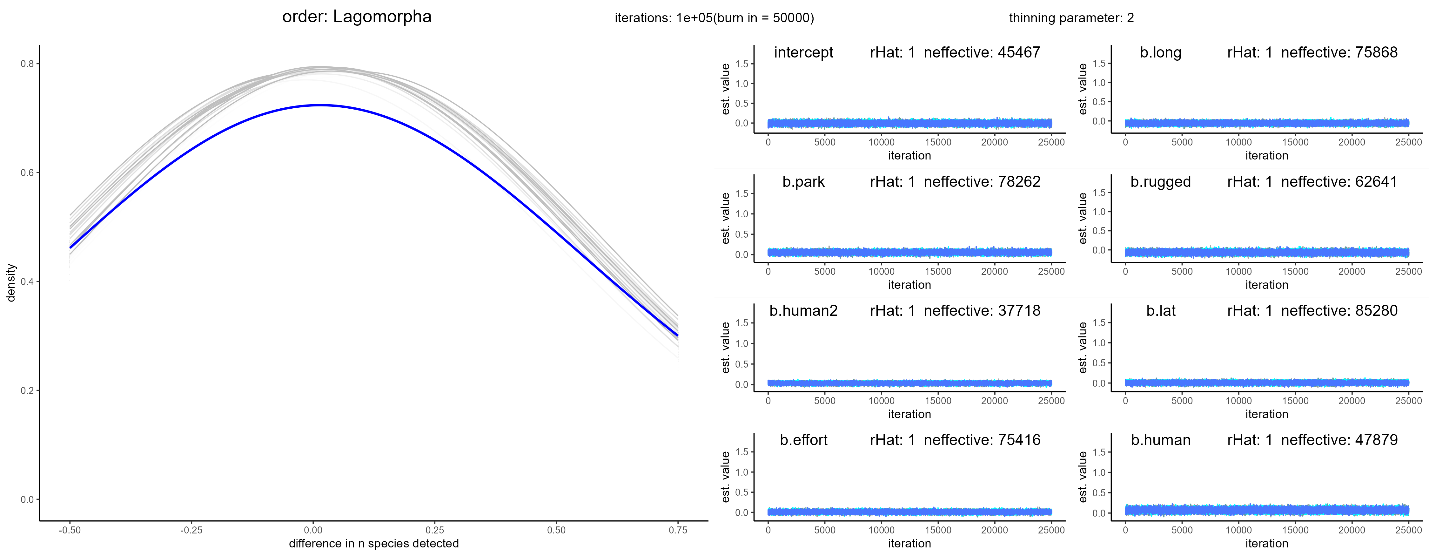


**Supplemental Figure 2.5**. Model diagnostics for the Mammalia richness model. The posterior predictive plot (left) compares the observed density of values (blue) against the predicted density of values using the estimated model coefficients and the training dataset. Trace plots (right) show mixing of MCMC chains.
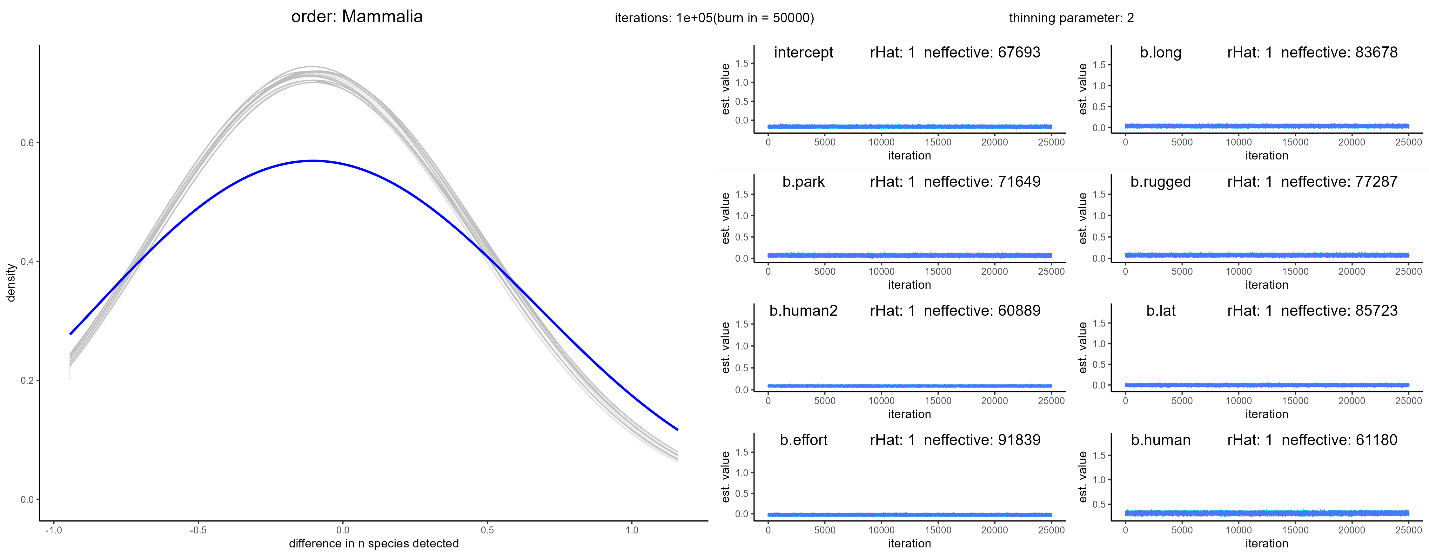


**Supplemental Figure 2.6**. Model diagnostics for the Rodentia richness model. The posterior predictive plot (left) compares the observed density of values (blue) against the predicted density of values using the estimated model coefficients and the training dataset. Trace plots (right) show mixing of MCMC chains.
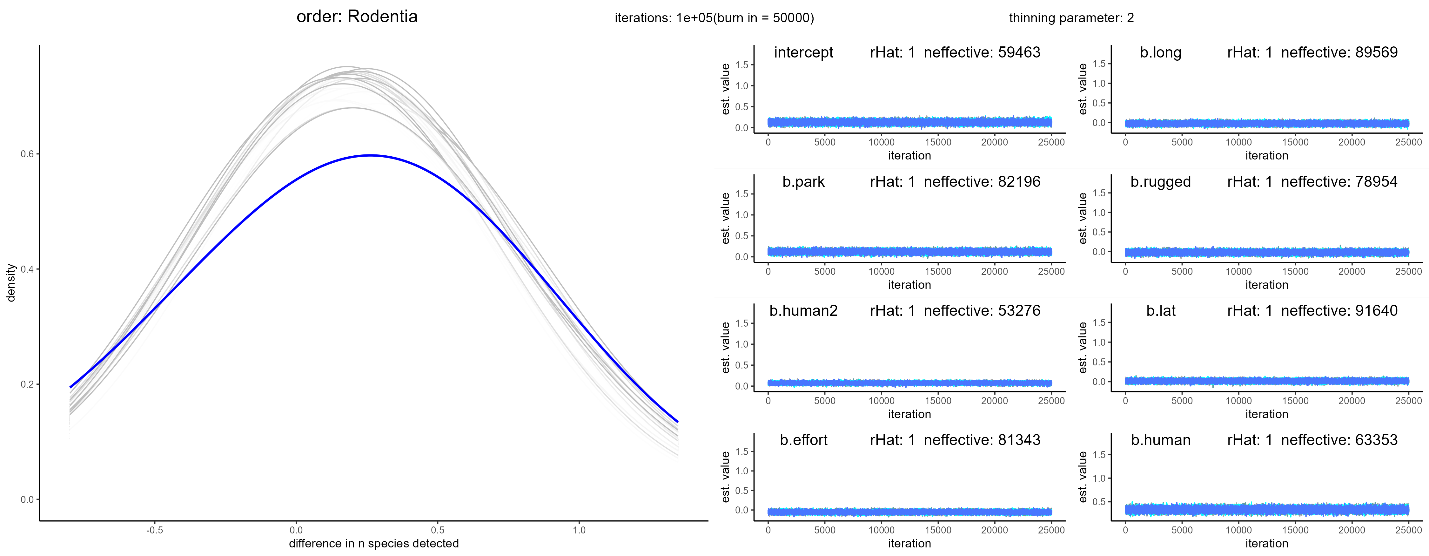


**Supplemental Figure 2.7**. Model diagnostics for the Artiodactyla species pool model. The posterior predictive plot (left) compares the observed density of values (blue) against the predicted density of values using the estimated model coefficients and the training dataset. Trace plots (right) show mixing of MCMC chains.
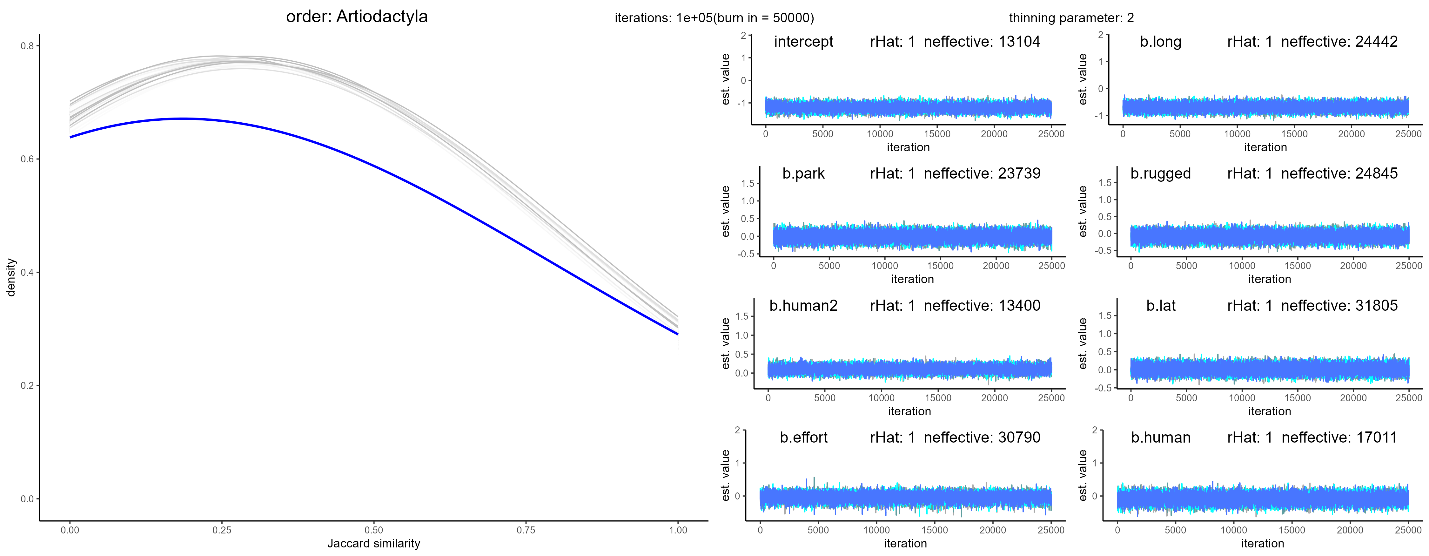


**Supplemental Figure 2.8**. Model diagnostics for the Carnivora species pool model. The posterior predictive plot (left) compares the observed density of values (blue) against the predicted density of values using the estimated model coefficients and the training dataset. Trace plots (right) show mixing of MCMC chains.
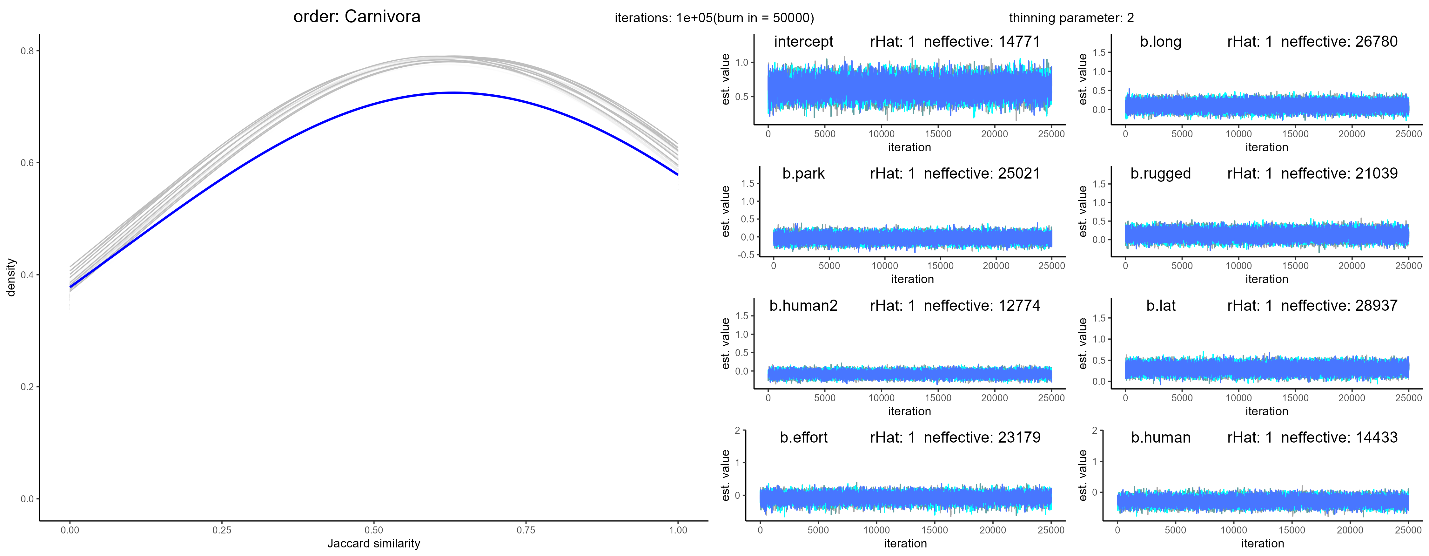


**Supplemental Figure 2.9**. Model diagnostics for the Chiroptera species pool model. The posterior predictive plot (left) compares the observed density of values (blue) against the predicted density of values using the estimated model coefficients and the training dataset. Trace plots (right) show mixing of MCMC chains.


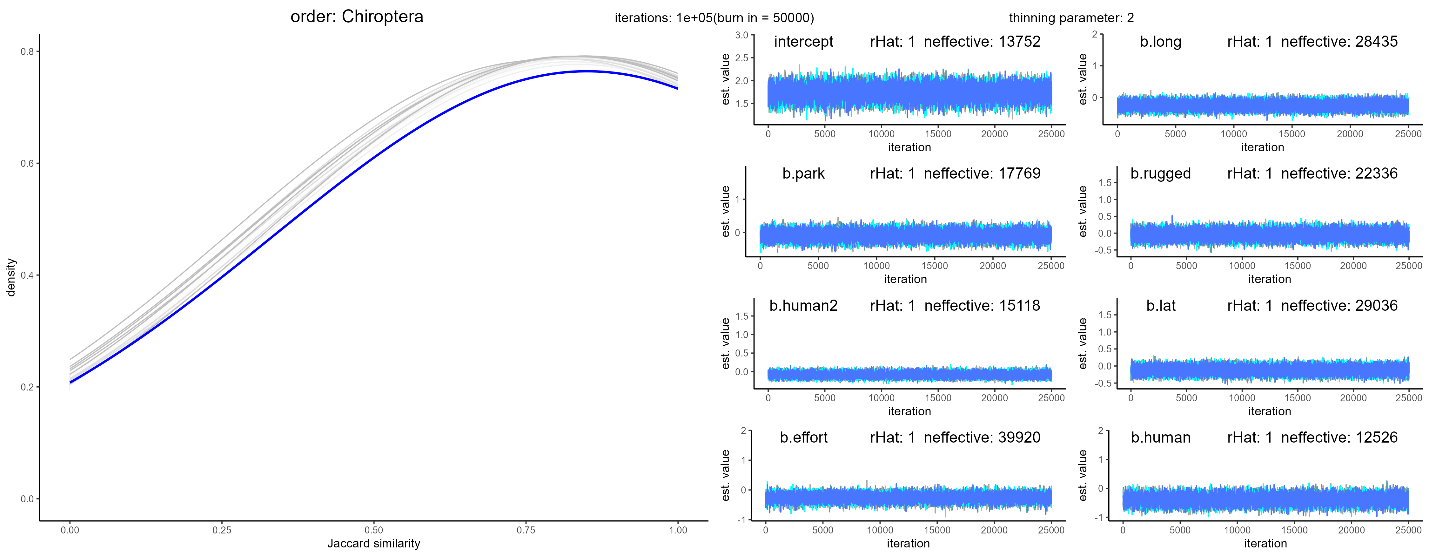


**Supplemental Figure 2.10**. Model diagnostics for the Lagomorpha species pool model. The posterior predictive plot (left) compares the observed density of values (blue) against the predicted density of values using the estimated model coefficients and the training dataset. Trace plots (right) show mixing of MCMC chains.
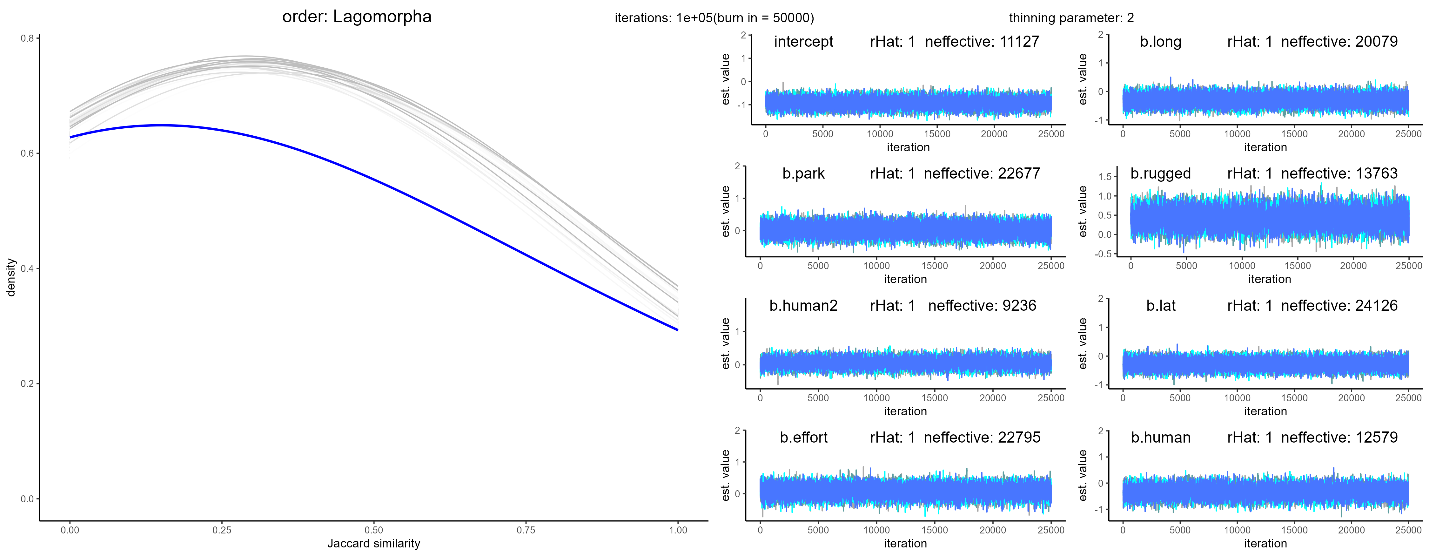


**Supplemental Figure 2.11**. Model diagnostics for the Mammalia species pool model. The posterior predictive plot (left) compares the observed density of values (blue) against the predicted density of values using the estimated model coefficients and the training dataset. Trace plots (right) show mixing of MCMC chains.
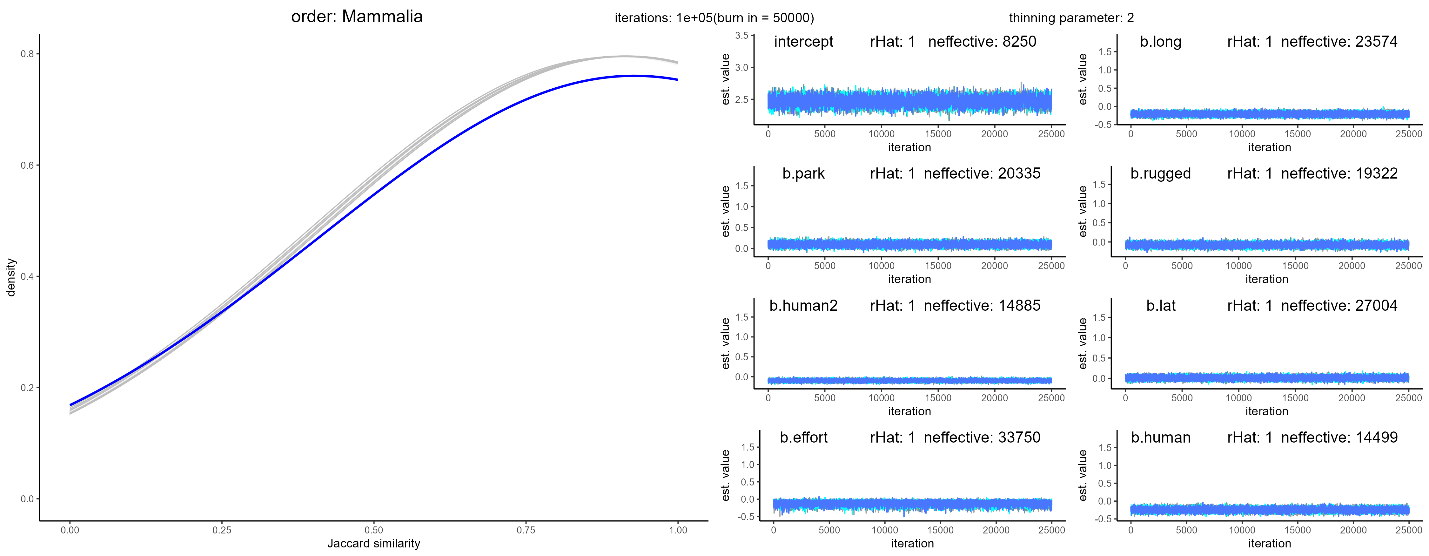


**Supplemental Figure 2.12**. Model diagnostics for the Rodentia species pool model. The posterior predictive plot (left) compares the observed density of values (blue) against the predicted density of values using the estimated model coefficients and the training dataset. Trace plots (right) show mixing of MCMC chains.
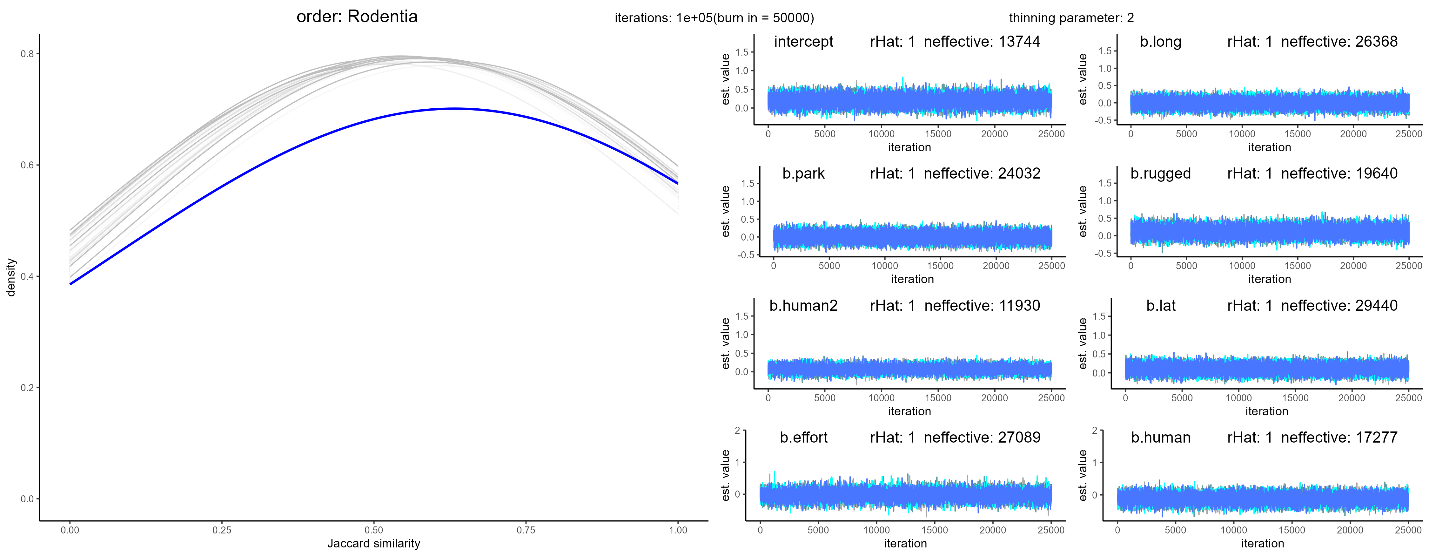

Supplement: Supplementary file 2 — Data S2. [file ECE3-15-e71805-s003.docx]
