# Supplementary material for: iNaturalist and Structured Mammal Surveys Reflect Similar Species Richness but Capture Different Species Pools Across the United States
Source: Ecol Evol. 2025 Jul 20;15(7):e71805. doi: 10.1002/ece3.71805 (PMC12276820; doi:10.1002/ece3.71805)
Supplement: Supplementary file 3 — Data S3. [file ECE3-15-e71805-s002.docx]

**Supplemental Table 3.1** Number of observations of each species in sampled grid cells from iNaturalist and structured surveys, and the number of grid cells with observations of each species from each survey method. Values within parentheses are the proportion of the total survey-specific dataset comprised of observations of the select species, or the proportion of grid cells with observations of the species from each survey method. Non-research grade iNaturalist observations were removed. Research-grade observations of non-native (i.e., captive) or non-terrestrial species were retained in the dataset.

| **Order** | **Species** | **Number of observations in iNaturalist** | **Number of observations in structured surveys** | **Number of sample cells with iNaturalist observations** | **Number of sample cells with structured observations** |
| --- | --- | --- | --- | --- | --- |
| Artiodactyla | *Alces alces* | 728 (1.07%) | 232 (0.05%) | 64 (6.99%) | 12 (1.31%) |
|  | *Ammotragus lervia* | 29 (0.04%) | 0 (0%) | 2 (0.22%) | 0 (0%) |
|  | *Antilocapra americana* | 378 (0.56%) | 187 (0.04%) | 86 (9.39%) | 8 (0.87%) |
|  | *Axis axis* | 1 (<1%) | 0 (0%) | 1 (0.11%) | 0 (0%) |
|  | *Balaenoptera acutorostrata* | 10 (0.01%) | 0 (0%) | 3 (0.33%) | 0 (0%) |
|  | *Balaenoptera physalus* | 5 (0.01%) | 0 (0%) | 3 (0.33%) | 0 (0%) |
|  | *Bison bison* | 126 (0.19%) | 918 (0.19%) | 10 (1.09%) | 6 (0.66%) |
|  | *Boselaphus tragocamelus* | 24 (0.04%) | 95 (0.02%) | 3 (0.33%) | 2 (0.22%) |
|  | *Capra aegagrus* | 1 (<1%) | 0 (0%) | 1 (0.11%) | 0 (0%) |
|  | *Cervus canadensis* | 1064 (1.57%) | 4973 (1.04%) | 111 (12.12%) | 26 (2.84%) |
|  | *Cervus elaphus* | 1 (<1%) | 0 (0%) | 1 (0.11%) | 0 (0%) |
|  | *Dama dama* | 2 (<1%) | 0 (0%) | 2 (0.22%) | 0 (0%) |
|  | *Delphinapterus leucas* | 3 (<1%) | 0 (0%) | 1 (0.11%) | 0 (0%) |
|  | *Delphinus delphis* | 13 (0.02%) | 0 (0%) | 5 (0.55%) | 0 (0%) |
|  | *Eschrichtius robustus* | 8 (0.01%) | 0 (0%) | 4 (0.44%) | 0 (0%) |
|  | *Eubalaena glacialis* | 3 (<1%) | 0 (0%) | 2 (0.22%) | 0 (0%) |
|  | *Globicephala melas* | 1 (<1%) | 0 (0%) | 1 (0.11%) | 0 (0%) |
|  | *Megaptera novaeangliae* | 38 (0.06%) | 0 (0%) | 11 (1.2%) | 0 (0%) |
|  | *Odocoileus hemionus* | 2351 (3.46%) | 14531 (3.04%) | 267 (29.15%) | 69 (7.53%) |
|  | *Odocoileus virginianus* | 6209 (9.14%) | 90126 (18.88%) | 296 (32.31%) | 180 (19.65%) |
|  | *Orcinus orca* | 50 (0.07%) | 0 (0%) | 14 (1.53%) | 0 (0%) |
|  | *Oreamnos americanus* | 114 (0.17%) | 0 (0%) | 31 (3.38%) | 0 (0%) |
|  | *Oryx gazella* | 3 (<1%) | 0 (0%) | 2 (0.22%) | 0 (0%) |
|  | *Ovis canadensis* | 368 (0.54%) | 0 (0%) | 70 (7.64%) | 0 (0%) |
|  | *Pecari tajacu* | 324 (0.48%) | 1010 (0.21%) | 37 (4.04%) | 11 (1.2%) |
|  | *Phocoena phocoena* | 27 (0.04%) | 0 (0%) | 8 (0.87%) | 0 (0%) |
|  | *Phocoenoides dalli* | 1 (<1%) | 0 (0%) | 1 (0.11%) | 0 (0%) |
|  | *Physeter macrocephalus* | 1 (<1%) | 0 (0%) | 1 (0.11%) | 0 (0%) |
|  | *Sus scrofa* | 141 (0.21%) | 6595 (1.38%) | 44 (4.8%) | 36 (3.93%) |
|  | *Tursiops truncatus* | 89 (0.13%) | 0 (0%) | 18 (1.97%) | 0 (0%) |
| Carnivora | *Arctocephalus townsendi* | 1 (<1%) | 0 (0%) | 1 (0.11%) | 0 (0%) |
|  | *Bassariscus astutus* | 59 (0.09%) | 213 (0.04%) | 22 (2.4%) | 7 (0.76%) |
|  | *Canis familiaris* | 34 (0.05%) | 5247 (1.1%) | 28 (3.06%) | 151 (16.48%) |
|  | *Canis latrans* | 2270 (3.34%) | 13943 (2.92%) | 333 (36.35%) | 201 (21.94%) |
|  | *Canis lupus* | 5 (0.01%) | 122 (0.03%) | 5 (0.55%) | 7 (0.76%) |
|  | *Canis rufus* | 17 (0.03%) | 105 (0.02%) | 4 (0.44%) | 5 (0.55%) |
|  | *Conepatus leuconotus* | 12 (0.02%) | 17 (<1%) | 11 (1.2%) | 4 (0.44%) |
|  | *Enhydra lutris* | 5 (0.01%) | 0 (0%) | 4 (0.44%) | 0 (0%) |
|  | *Eumetopias jubatus* | 73 (0.11%) | 0 (0%) | 16 (1.75%) | 0 (0%) |
|  | *Felis catus* | 924 (1.36%) | 2088 (0.44%) | 120 (13.1%) | 66 (7.21%) |
|  | *Gulo gulo* | 1 (<1%) | 14889 (3.12%) | 1 (0.11%) | 5 (0.55%) |
|  | *Halichoerus grypus* | 25 (0.04%) | 0 (0%) | 7 (0.76%) | 0 (0%) |
|  | *Lontra canadensis* | 562 (0.83%) | 70 (0.01%) | 147 (16.05%) | 17 (1.86%) |
|  | *Lynx rufus* | 690 (1.02%) | 2687 (0.56%) | 183 (19.98%) | 130 (14.19%) |
|  | *Martes americana* | 2 (<1%) | 36 (0.01%) | 2 (0.22%) | 7 (0.76%) |
|  | *Mephitis macroura* | 10 (0.01%) | 5 (<1%) | 7 (0.76%) | 4 (0.44%) |
|  | *Mephitis mephitis* | 533 (0.78%) | 1375 (0.29%) | 144 (15.72%) | 96 (10.48%) |
|  | *Mirounga angustirostris* | 4 (0.01%) | 0 (0%) | 2 (0.22%) | 0 (0%) |
|  | *Nasua narica* | 224 (0.33%) | 58 (0.01%) | 16 (1.75%) | 2 (0.22%) |
|  | *Pagophilus groenlandicus* | 2 (<1%) | 0 (0%) | 1 (0.11%) | 0 (0%) |
|  | *Phoca vitulina* | 133 (0.2%) | 0 (0%) | 24 (2.62%) | 0 (0%) |
|  | *Procyon lotor* | 2902 (4.27%) | 38917 (8.15%) | 296 (32.31%) | 178 (19.43%) |
|  | *Puma concolor* | 87 (0.13%) | 419 (0.09%) | 52 (5.68%) | 24 (2.62%) |
|  | *Spilogale gracilis* | 20 (0.03%) | 102 (0.02%) | 10 (1.09%) | 10 (1.09%) |
|  | *Spilogale putorius* | 2 (<1%) | 4 (<1%) | 2 (0.22%) | 2 (0.22%) |
|  | *Taxidea taxus* | 62 (0.09%) | 68 (0.01%) | 38 (4.15%) | 15 (1.64%) |
|  | *Urocyon cinereoargenteus* | 423 (0.62%) | 3754 (0.79%) | 128 (13.97%) | 60 (6.55%) |
|  | *Ursus americanus* | 688 (1.01%) | 12885 (2.7%) | 205 (22.38%) | 81 (8.84%) |
|  | *Ursus arctos* | 2 (<1%) | 11 (<1%) | 2 (0.22%) | 2 (0.22%) |
|  | *Vulpes macrotis* | 19 (0.03%) | 90 (0.02%) | 14 (1.53%) | 4 (0.44%) |
|  | *Vulpes velox* | 3 (<1%) | 24 (0.01%) | 3 (0.33%) | 3 (0.33%) |
|  | *Vulpes vulpes* | 1499 (2.21%) | 10208 (2.14%) | 176 (19.21%) | 86 (9.39%) |
|  | *Zalophus californianus* | 139 (0.2%) | 0 (0%) | 14 (1.53%) | 0 (0%) |
| Chiroptera | *Mustela erminea* | 0 (0%) | 14 (<1%) | 0 (0%) | 8 (0.87%) |
|  | *Neogale frenata* | 0 (0%) | 56 (0.01%) | 0 (0%) | 20 (2.18%) |
|  | *Dipodomys ingens* | 0 (0%) | 24 (0.01%) | 0 (0%) | 1 (0.11%) |
|  | *Neogale vison* | 0 (0%) | 58 (0.01%) | 0 (0%) | 19 (2.07%) |
|  | *Sylvilagus obscurus* | 0 (0%) | 8 (<1%) | 0 (0%) | 2 (0.22%) |
|  | *Lariscus insignis* | 0 (0%) | 1 (<1%) | 0 (0%) | 1 (0.11%) |
|  | *Didelphis marsupialis* | 0 (0%) | 32 (0.01%) | 0 (0%) | 1 (0.11%) |
|  | *Eumops floridanus* | 0 (0%) | 245 (0.05%) | 0 (0%) | 9 (0.98%) |
|  | *Lasiurus seminolus* | 0 (0%) | 1876 (0.39%) | 0 (0%) | 13 (1.42%) |
|  | *Eptesicus fuscus* | 0 (0%) | 13175 (2.76%) | 0 (0%) | 510 (55.68%) |
|  | *Tadarida brasiliensis* | 0 (0%) | 30159 (6.32%) | 0 (0%) | 349 (38.1%) |
|  | *Nycticeius humeralis* | 0 (0%) | 9550 (2%) | 0 (0%) | 281 (30.68%) |
|  | *Lasiurus intermedius* | 0 (0%) | 6968 (1.46%) | 0 (0%) | 29 (3.17%) |
|  | *Antrozous pallidus* | 0 (0%) | 1351 (0.28%) | 0 (0%) | 178 (19.43%) |
|  | *Lasionycteris noctivagans* | 0 (0%) | 9916 (2.08%) | 0 (0%) | 533 (58.19%) |
|  | *Euderma maculatum* | 0 (0%) | 711 (0.15%) | 0 (0%) | 98 (10.7%) |
|  | *Lasiurus borealis* | 0 (0%) | 5331 (1.12%) | 0 (0%) | 112 (12.23%) |
|  | *Lasiurus cinereus* | 0 (0%) | 7571 (1.59%) | 0 (0%) | 514 (56.11%) |
|  | *Corynorhinus townsendii* | 0 (0%) | 867 (0.18%) | 0 (0%) | 173 (18.89%) |
|  | *Lasiurus xanthinus* | 0 (0%) | 200 (0.04%) | 0 (0%) | 35 (3.82%) |
|  | *Myotis lucifugus* | 0 (0%) | 14619 (3.06%) | 0 (0%) | 409 (44.65%) |
|  | *Myotis leibii* | 0 (0%) | 541 (0.11%) | 0 (0%) | 16 (1.75%) |
|  | *Myotis ciliolabrum* | 0 (0%) | 4545 (0.95%) | 0 (0%) | 277 (30.24%) |
|  | *Lasiurus frantzii* | 0 (0%) | 555 (0.12%) | 0 (0%) | 79 (8.62%) |
|  | *Idionycteris phyllotis* | 0 (0%) | 82 (0.02%) | 0 (0%) | 15 (1.64%) |
|  | *Myotis septentrionalis* | 0 (0%) | 54 (0.01%) | 0 (0%) | 16 (1.75%) |
|  | *Myotis grisescens* | 0 (0%) | 184 (0.04%) | 0 (0%) | 10 (1.09%) |
|  | *Myotis sodalis* | 0 (0%) | 43 (0.01%) | 0 (0%) | 8 (0.87%) |
|  | *Corynorhinus rafinesquii* | 0 (0%) | 28 (0.01%) | 0 (0%) | 10 (1.09%) |
|  | *Choeronycteris mexicana* | 0 (0%) | 4 (<1%) | 0 (0%) | 1 (0.11%) |
| Cingulata | *Dasypus novemcinctus* | 470 (0.69%) | 2990 (0.63%) | 85 (9.28%) | 49 (5.35%) |
| Didelphimorphia | *Didelphis virginiana* | 1134 (1.67%) | 7487 (1.57%) | 186 (20.31%) | 134 (14.63%) |
| Lagomorpha | *Lepus alleni* | 19 (0.03%) | 4 (<1%) | 8 (0.87%) | 1 (0.11%) |
|  | *Lepus americanus* | 100 (0.15%) | 517 (0.11%) | 42 (4.59%) | 24 (2.62%) |
|  | *Lepus californicus* | 405 (0.6%) | 420 (0.09%) | 88 (9.61%) | 21 (2.29%) |
|  | *Lepus townsendii* | 5 (0.01%) | 133 (0.03%) | 4 (0.44%) | 6 (0.66%) |
|  | *Ochotona princeps* | 385 (0.57%) | 0 (0%) | 43 (4.69%) | 0 (0%) |
|  | *Oryctolagus cuniculus* | 14 (0.02%) | 0 (0%) | 10 (1.09%) | 0 (0%) |
|  | *Sylvilagus aquaticus* | 26 (0.04%) | 7 (<1%) | 10 (1.09%) | 1 (0.11%) |
|  | *Sylvilagus audubonii* | 1376 (2.03%) | 3503 (0.73%) | 88 (9.61%) | 11 (1.2%) |
|  | *Sylvilagus bachmani* | 154 (0.23%) | 97 (0.02%) | 14 (1.53%) | 4 (0.44%) |
|  | *Sylvilagus floridanus* | 5881 (8.66%) | 6658 (1.39%) | 187 (20.41%) | 99 (10.81%) |
|  | *Sylvilagus nuttallii* | 84 (0.12%) | 140 (0.03%) | 38 (4.15%) | 7 (0.76%) |
|  | *Sylvilagus palustris* | 261 (0.38%) | 136 (0.03%) | 17 (1.86%) | 7 (0.76%) |
|  | *Sylvilagus transitionalis* | 1 (<1%) | 0 (0%) | 1 (0.11%) | 0 (0%) |
| Rodentia | *Ammospermophilus harrisii* | 129 (0.19%) | 14 (<1%) | 16 (1.75%) | 3 (0.33%) |
|  | *Ammospermophilus leucurus* | 680 (1%) | 370 (0.08%) | 29 (3.17%) | 4 (0.44%) |
|  | *Ammospermophilus nelsoni* | 60 (0.09%) | 0 (0%) | 4 (0.44%) | 0 (0%) |
|  | *Aplodontia rufa* | 14 (0.02%) | 6 (<1%) | 9 (0.98%) | 2 (0.22%) |
|  | *Callospermophilus lateralis* | 967 (1.42%) | 86 (0.02%) | 89 (9.72%) | 6 (0.66%) |
|  | *Callospermophilus saturatus* | 254 (0.37%) | 0 (0%) | 16 (1.75%) | 0 (0%) |
|  | *Castor canadensis* | 1818 (2.68%) | 742 (0.16%) | 247 (26.97%) | 15 (1.64%) |
|  | *Cratogeomys castanops* | 1 (<1%) | 0 (0%) | 1 (0.11%) | 0 (0%) |
|  | *Cynomys gunnisoni* | 109 (0.16%) | 0 (0%) | 21 (2.29%) | 0 (0%) |
|  | *Cynomys leucurus* | 8 (0.01%) | 0 (0%) | 1 (0.11%) | 0 (0%) |
|  | *Cynomys ludovicianus* | 351 (0.52%) | 360 (0.08%) | 19 (2.07%) | 3 (0.33%) |
|  | *Dipodomys spectabilis* | 1 (<1%) | 0 (0%) | 1 (0.11%) | 0 (0%) |
|  | *Erethizon dorsatum* | 305 (0.45%) | 249 (0.05%) | 99 (10.81%) | 32 (3.49%) |
|  | *Geomys bursarius* | 1 (<1%) | 0 (0%) | 1 (0.11%) | 0 (0%) |
|  | *Geomys personatus* | 2 (<1%) | 0 (0%) | 1 (0.11%) | 0 (0%) |
|  | *Glaucomys sabrinus* | 7 (0.01%) | 254 (0.05%) | 7 (0.76%) | 23 (2.51%) |
|  | *Ictidomys tridecemlineatus* | 28 (0.04%) | 1 (<1%) | 17 (1.86%) | 1 (0.11%) |
|  | *Marmota caligata* | 274 (0.4%) | 0 (0%) | 9 (0.98%) | 0 (0%) |
|  | *Marmota flaviventris* | 590 (0.87%) | 0 (0%) | 67 (7.31%) | 0 (0%) |
|  | *Marmota monax* | 1223 (1.8%) | 414 (0.09%) | 120 (13.1%) | 30 (3.28%) |
|  | *Marmota olympus* | 52 (0.08%) | 0 (0%) | 3 (0.33%) | 0 (0%) |
|  | *Myocastor coypus* | 308 (0.45%) | 0 (0%) | 24 (2.62%) | 0 (0%) |
|  | *Neofiber alleni* | 3 (<1%) | 0 (0%) | 2 (0.22%) | 0 (0%) |
|  | *Neotamias minimus* | 48 (0.07%) | 3 (<1%) | 15 (1.64%) | 2 (0.22%) |
|  | *Neotoma albigula* | 54 (0.08%) | 4 (<1%) | 14 (1.53%) | 2 (0.22%) |
|  | *Neotoma bryanti* | 1 (<1%) | 0 (0%) | 1 (0.11%) | 0 (0%) |
|  | *Neotoma cinerea* | 5 (0.01%) | 26 (0.01%) | 5 (0.55%) | 2 (0.22%) |
|  | *Neotoma floridana* | 19 (0.03%) | 36 (0.01%) | 2 (0.22%) | 5 (0.55%) |
|  | *Neotoma fuscipes* | 28 (0.04%) | 7 (<1%) | 3 (0.33%) | 1 (0.11%) |
|  | *Neotoma lepida* | 7 (0.01%) | 53 (0.01%) | 7 (0.76%) | 2 (0.22%) |
|  | *Neotoma macrotis* | 2 (<1%) | 0 (0%) | 2 (0.22%) | 0 (0%) |
|  | *Neotoma magister* | 1 (<1%) | 0 (0%) | 1 (0.11%) | 0 (0%) |
|  | *Neotoma mexicana* | 3 (<1%) | 11 (<1%) | 3 (0.33%) | 2 (0.22%) |
|  | *Ondatra zibethicus* | 976 (1.44%) | 22 (<1%) | 135 (14.74%) | 3 (0.33%) |
|  | *Otospermophilus beecheyi* | 1075 (1.58%) | 1063 (0.22%) | 73 (7.97%) | 9 (0.98%) |
|  | *Otospermophilus variegatus* | 429 (0.63%) | 147 (0.03%) | 57 (6.22%) | 7 (0.76%) |
|  | *Rattus norvegicus* | 838 (1.23%) | 28 (0.01%) | 63 (6.88%) | 5 (0.55%) |
|  | *Rattus rattus* | 57 (0.08%) | 5 (<1%) | 29 (3.17%) | 1 (0.11%) |
|  | *Sciurus aberti* | 187 (0.28%) | 15 (<1%) | 26 (2.84%) | 2 (0.22%) |
|  | *Sciurus arizonensis* | 251 (0.37%) | 0 (0%) | 13 (1.42%) | 0 (0%) |
|  | *Sciurus carolinensis* | 14918 (21.96%) | 78112 (16.36%) | 228 (24.89%) | 129 (14.08%) |
|  | *Sciurus griseus* | 859 (1.26%) | 4444 (0.93%) | 41 (4.48%) | 18 (1.97%) |
|  | *Sciurus niger* | 2105 (3.1%) | 12988 (2.72%) | 112 (12.23%) | 52 (5.68%) |
|  | *Sigmodon arizonae* | 16 (0.02%) | 0 (0%) | 6 (0.66%) | 0 (0%) |
|  | *Sigmodon ochrognathus* | 4 (0.01%) | 0 (0%) | 3 (0.33%) | 0 (0%) |
|  | *Tamias striatus* | 2751 (4.05%) | 8009 (1.68%) | 135 (14.74%) | 64 (6.99%) |
|  | *Tamiasciurus douglasii* | 558 (0.82%) | 6411 (1.34%) | 84 (9.17%) | 20 (2.18%) |
|  | *Tamiasciurus hudsonicus* | 962 (1.42%) | 5375 (1.13%) | 123 (13.43%) | 43 (4.69%) |
|  | *Thomomys bottae* | 281 (0.41%) | 0 (0%) | 64 (6.99%) | 0 (0%) |
|  | *Thomomys bulbivorus* | 2 (<1%) | 0 (0%) | 1 (0.11%) | 0 (0%) |
|  | *Thomomys talpoides* | 4 (0.01%) | 0 (0%) | 3 (0.33%) | 0 (0%) |
|  | *Thomomys umbrinus* | 1 (<1%) | 0 (0%) | 1 (0.11%) | 0 (0%) |
|  | *Urocitellus armatus* | 15 (0.02%) | 0 (0%) | 5 (0.55%) | 0 (0%) |
|  | *Urocitellus beldingi* | 20 (0.03%) | 0 (0%) | 11 (1.2%) | 0 (0%) |
|  | *Urocitellus columbianus* | 30 (0.04%) | 0 (0%) | 7 (0.76%) | 0 (0%) |
|  | *Urocitellus elegans* | 67 (0.1%) | 0 (0%) | 11 (1.2%) | 0 (0%) |
|  | *Urocitellus richardsonii* | 4 (0.01%) | 0 (0%) | 2 (0.22%) | 0 (0%) |
|  | *Urocitellus townsendii* | 3 (<1%) | 0 (0%) | 2 (0.22%) | 0 (0%) |
|  | *Xerospermophilus tereticaudus* | 107 (0.16%) | 3 (<1%) | 13 (1.42%) | 2 (0.22%) |

**Table 3.2** Estimated model coefficients explaining the relationship between landscape and survey variables and various diversity indices across mammalian taxa. Bold values indicate that the credible interval does not contain zero, and the estimate is accordingly regarded as significant. Credible intervals are listed in parentheses.

|  |  | Intercept | Latitude | Longitude | Human population density | Human population density^2^ | Landscape  ruggedness | Protected lands | Survey Effort |
| --- | --- | --- | --- | --- | --- | --- | --- | --- | --- |
| Proportional difference in species richness | Mammalia | **-0.17 (-0.20 – -0.14)** | -0.00 (-0.03 – 0.02) | **0.04 (0.01 – 0.07)** | **0.32 (0.28 – 0.35)** | **0.09 (0.07 – 0.11)** | **0.07 (0.04 – 0.10)** | **0.07 (0.04 – 0.10)** | -0.02 (-0.05 – 0.01) |
|  | Carnivora | **-0.33 (-0.39 – -0.26)** | 0 (-0.06 – 0.06) | -0.05 (-0.10 – 0.01) | **0.23 (0.16 – 0.30)** | **0.08 (0.04 – 0.12)** | -0.06 (-0.12 – 0.01) | -0.02 (-0.07 – 0.04) | -0.01 (-0.07 – 0.04) |
|  | Artiodactyla | -0.01 (-0.06 – 0.04) | -0.01 (-0.05 – 0.04) | -0.05 (-0.09 – 0.00) | **0.17 (0.12 – 0.22)** | 0.00 (-0.03 – 0.03) | **0.04 (0.01 – 0.09)** | 0.03 (-0.02 – 0.08) | -0.01 (-0.05 – 0.03) |
|  | Rodentia | **0.13 (0.06 – 0.20)** | 0.02 (-0.04 – 0.08) | -0.02 (-0.09 – 0.04) | **0.34 (0.26 – 0.41)** | **0.07 (0.03 – 0.12)** | -0.02 (-0.09 – 0.05) | **0.12 (0.06 – 0.19)** | -0.05 (-0.11 – 0.01) |
|  | Lagomorpha | 0.00 (-0.07 – 0.06) | 0.01 (-0.05 – 0.06) | -0.06 (-0.12 – 0.01) | **0.07 (0.00 – 0.15)** | 0.03 (-0.01 – 0.08) | -0.06 (-0.14 – 0.01) | 0.05 (-0.01 – 0.12) | 0.01 (-0.04 – 0.06) |
|  | Chiroptera | **-0.63 (-0.68 – -0.57)** | **-0.06 (-0.11 – -0.01)** | **0.06 (0.01 – 0.11)** | 0.02 (-0.05 – 0.09) | 0.01 (-0.03 – 0.04) | 0.02 (-0.04 – 0.08) | -0.03 (-0.09 – 0.04) | **0.06 (0.01 – 0.10)** |
| Jaccard Dissimilarity between surveyed communities | Mammalia | **2.47 (2.34 – 2.60)** | 0.01 (-0.06 – 0.09) | **-0.21 (-0.29 – -0.13)** | **-0.24 (-0.34 – -0.14)** | **-0.10 (-0.15 – -0.05)** | -0.08 (-0.17 – 0.01) | **0.10 (0.01 – 0.19)** | **-0.11 (-0.23 – -0.03)** |
|  | Carnivora | **0.63 (0.42 – 0.84)** | **0.30 (0.13 – 0.47)** | 0.10 (-0.09 – 0.29) | **-0.28 (-0.51 – -0.06)** | -0.08 (-0.22 – 0.05) | 0.13 (-0.08 – 0.33) | -0.03 (-0.22 – 0.16) | -0.07 (-0.29 – 0.13) |
|  | Artiodactyla | **-1.21 (-1.47 – -0.95)** | 0.02 (-0.18 – 0.22) | **-0.69 (-0.91 – -0.47)** | -0.08 (-0.32 – 0.15) | 0.10 (-0.05 – 0.26) | -0.08 (-0.30 – 0.13) | -0.01 (-0.22 – 0.2) | -0.02 (-0.24 – 0.15) |
|  | Rodentia | 0.2 (-0.05 – 0.46) | 0.11 (-0.1 – 0.31) | -0.01 (-0.24 – 0.21) | -0.12 (-0.38 – 0.14) | 0.07 (-0.1 – 0.24) | 0.12 (-0.13 – 0.37) | 0 (-0.22 – 0.23) | -0.04 (-0.28 – 0.21) |
|  | Lagomorpha | **-0.92 (-1.29 – -0.55)** | -0.28 (-0.57 – 0.02) | -0.31 (-0.63 – 0.02) | -0.34 (-0.74 – 0.06) | 0.05 (-0.2 – 0.31) | **0.42 (0.02 – 0.83)** | 0.02 (-0.3 – 0.34) | 0.1 (-0.23 – 0.4) |
|  | Chiroptera | **1.74 (1.46 – 2.01)** | -0.12 (-0.32 – 0.08) | **-0.24 (-0.45 – -0.03)** | **-0.39 (-0.68 – -0.10)** | -0.09 (-0.21 – 0.04) | -0.05 (-0.28 – 0.18) | -0.08 (-0.33 – 0.17) | **-0.26 (-0.46 – -0.05)** |
