# Supplementary material for: iNaturalist and Structured Mammal Surveys Reflect Similar Species Richness but Capture Different Species Pools Across the United States
Source: Ecol Evol. 2025 Jul 20;15(7):e71805. doi: 10.1002/ece3.71805 (PMC12276820; doi:10.1002/ece3.71805)

**Supplemental Figure 4.1.** Enlarged predictive map for similarity in Artiodactyla species richness between iNaturalist and camera trap data. The legend of the color scale is depicted in Figure 3 of the main text.
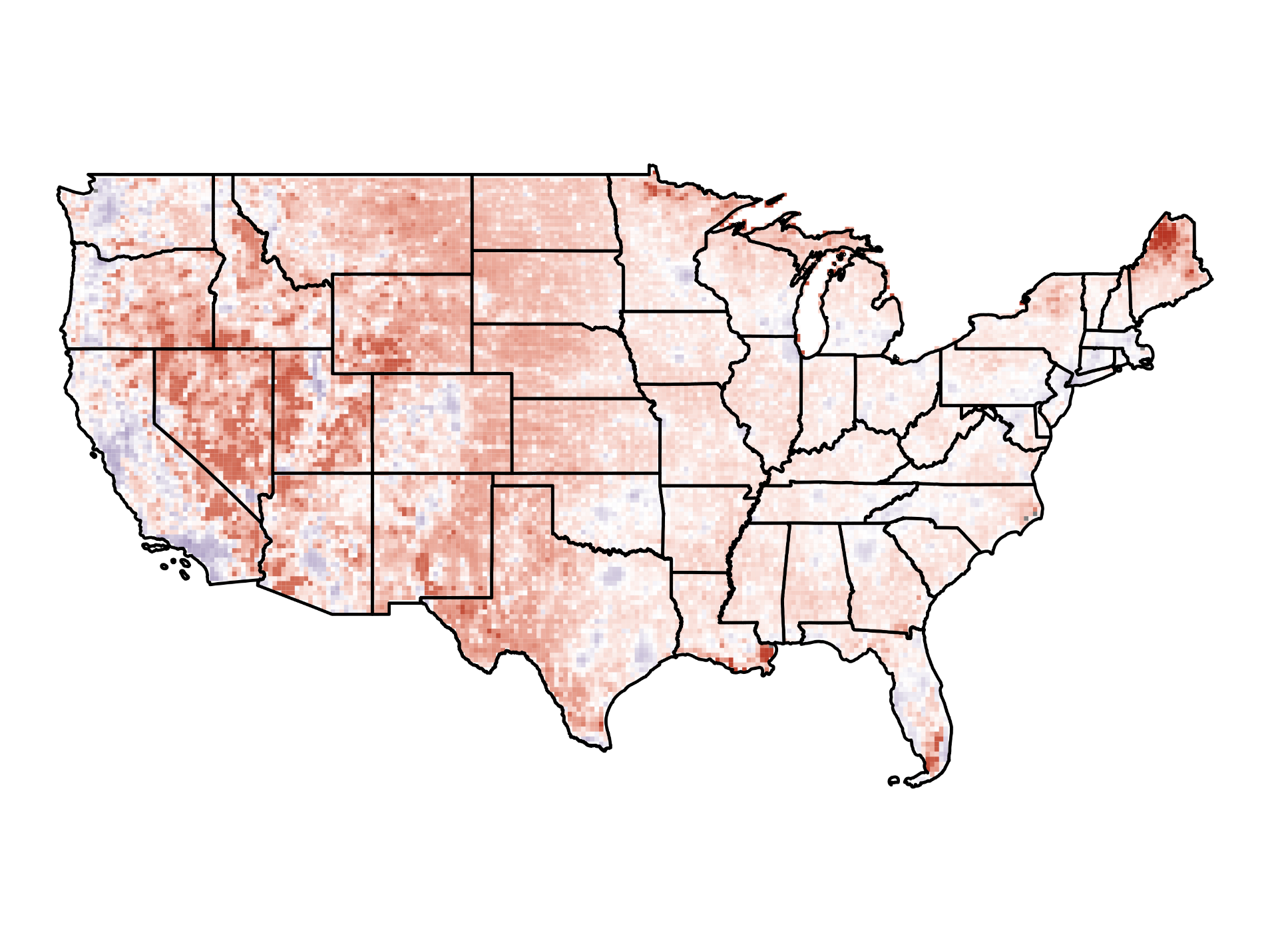


**Supplemental Figure 4.2.** Enlarged predictive map for similarity in Carnivora species richness between iNaturalist and camera trap data. The legend of the color scale is depicted in Figure 3 of the main text.**
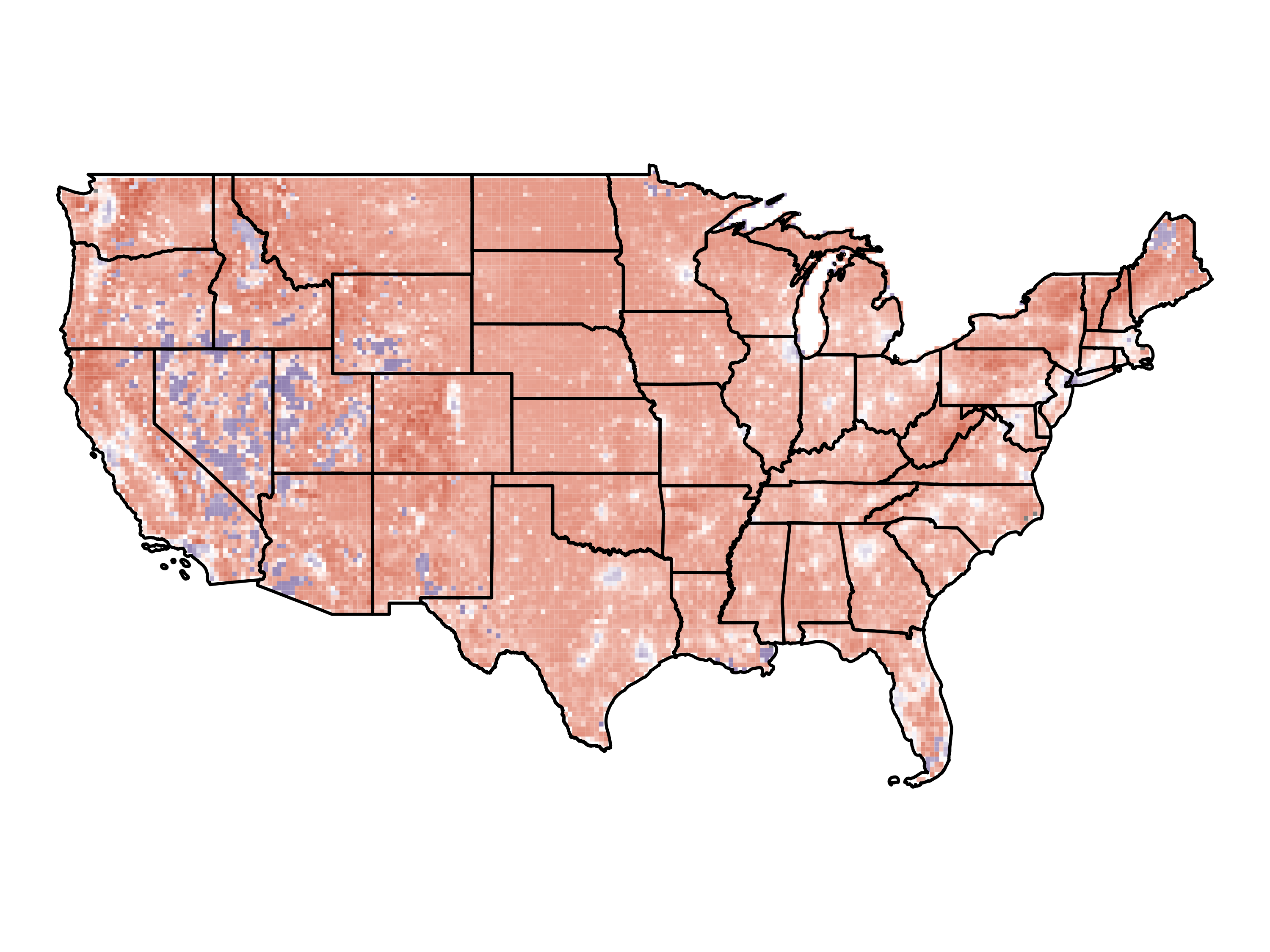
**

**Supplemental Figure 4.3.** Enlarged predictive map for similarity in Chiroptera species richness between iNaturalist and camera trap data. The legend of the color scale is depicted in Figure 3 of the main text.
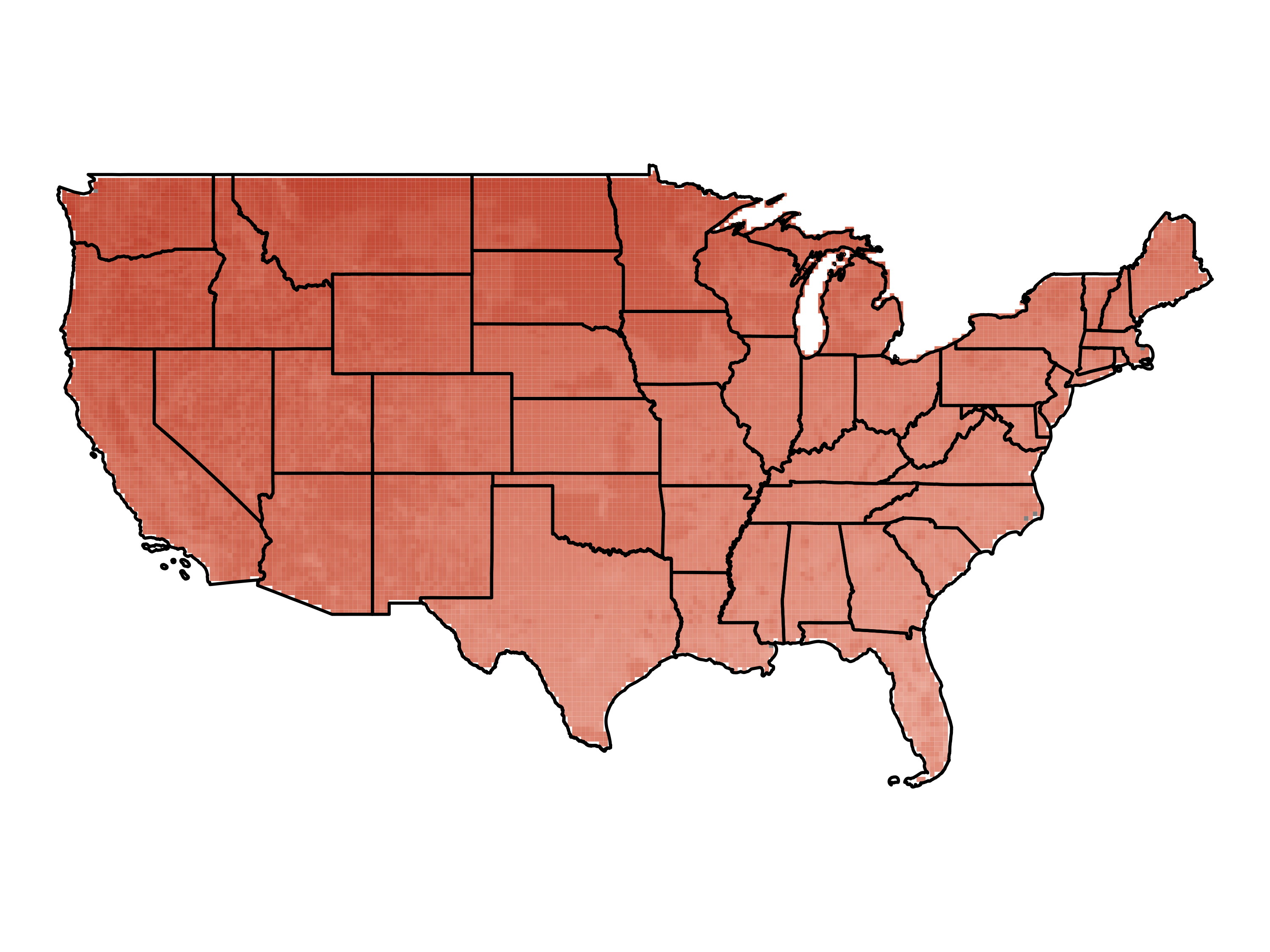


**Supplemental Figure 4.4.** Enlarged predictive map for similarity in Lagomorpha species richness between iNaturalist and camera trap data. The legend of the color scale is depicted in Figure 3 of the main text.
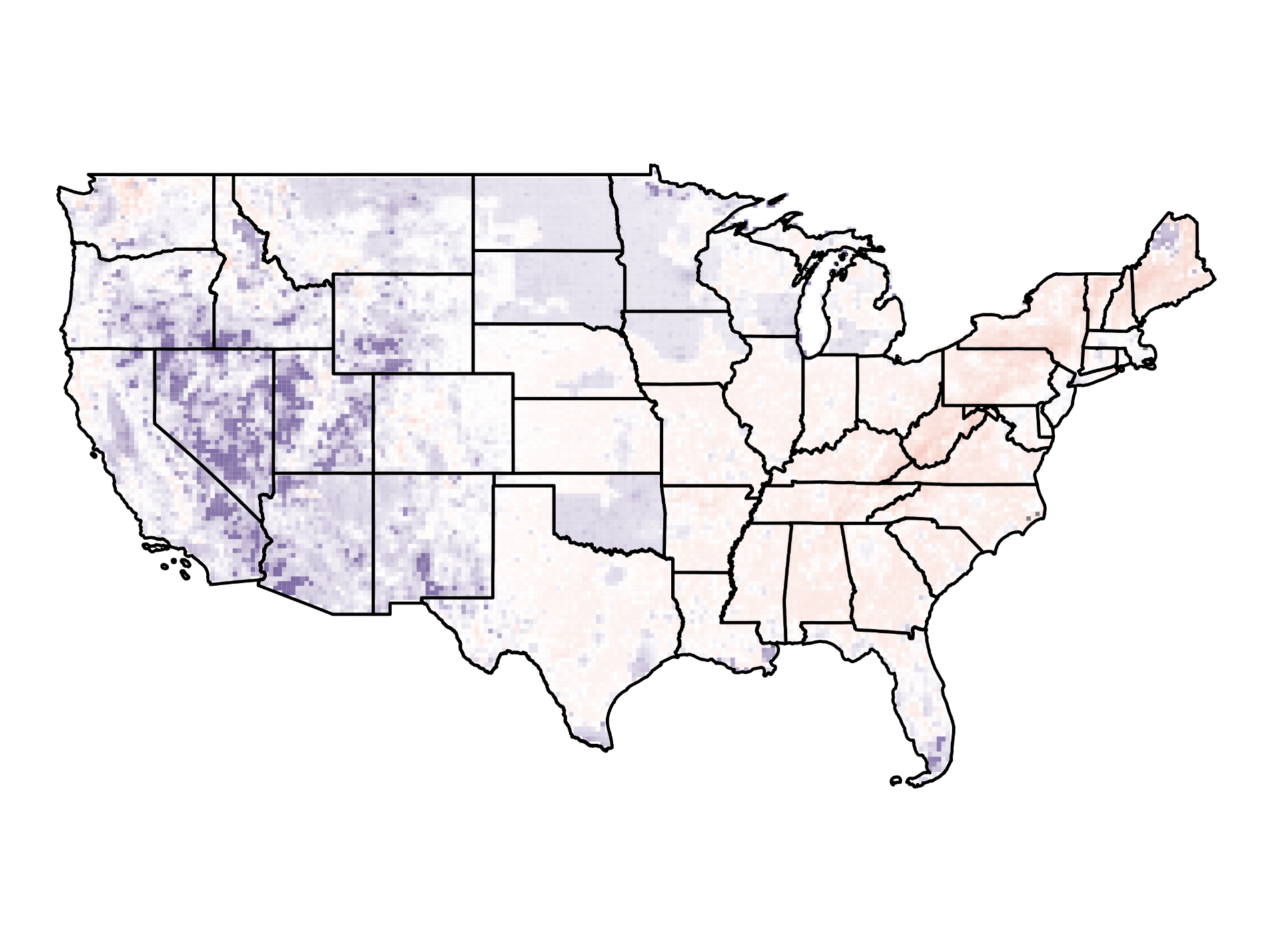


**Supplemental Figure 4.5.** Enlarged predictive map for similarity in Mammalia species richness between iNaturalist and camera trap data. The legend of the color scale is depicted in Figure 3 of the main text.
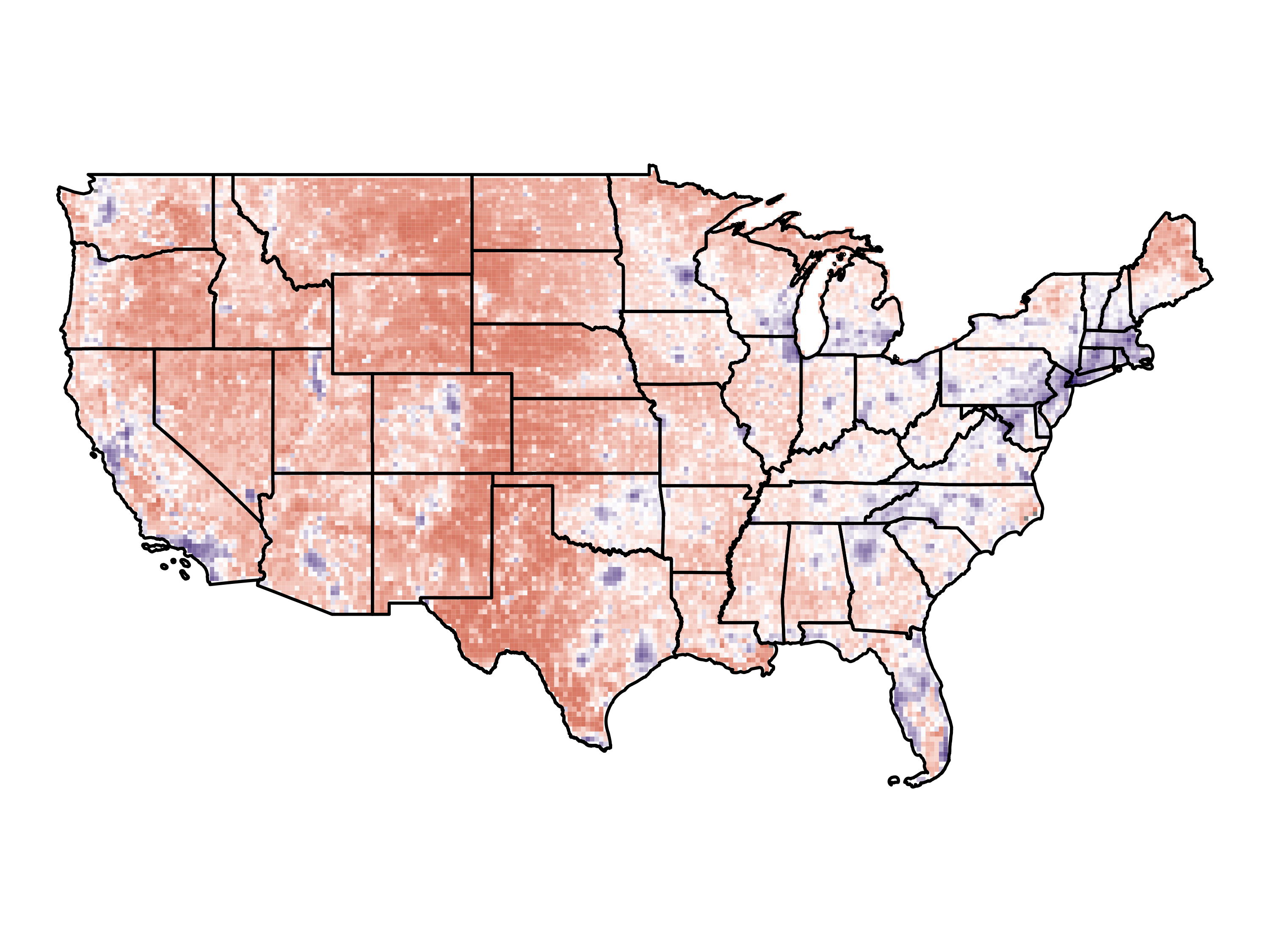


**Supplemental Figure 4.6.** Enlarged predictive map for similarity in Rodentia species richness between iNaturalist and camera trap data. The legend of the color scale is depicted in Figure 3 of the main text.
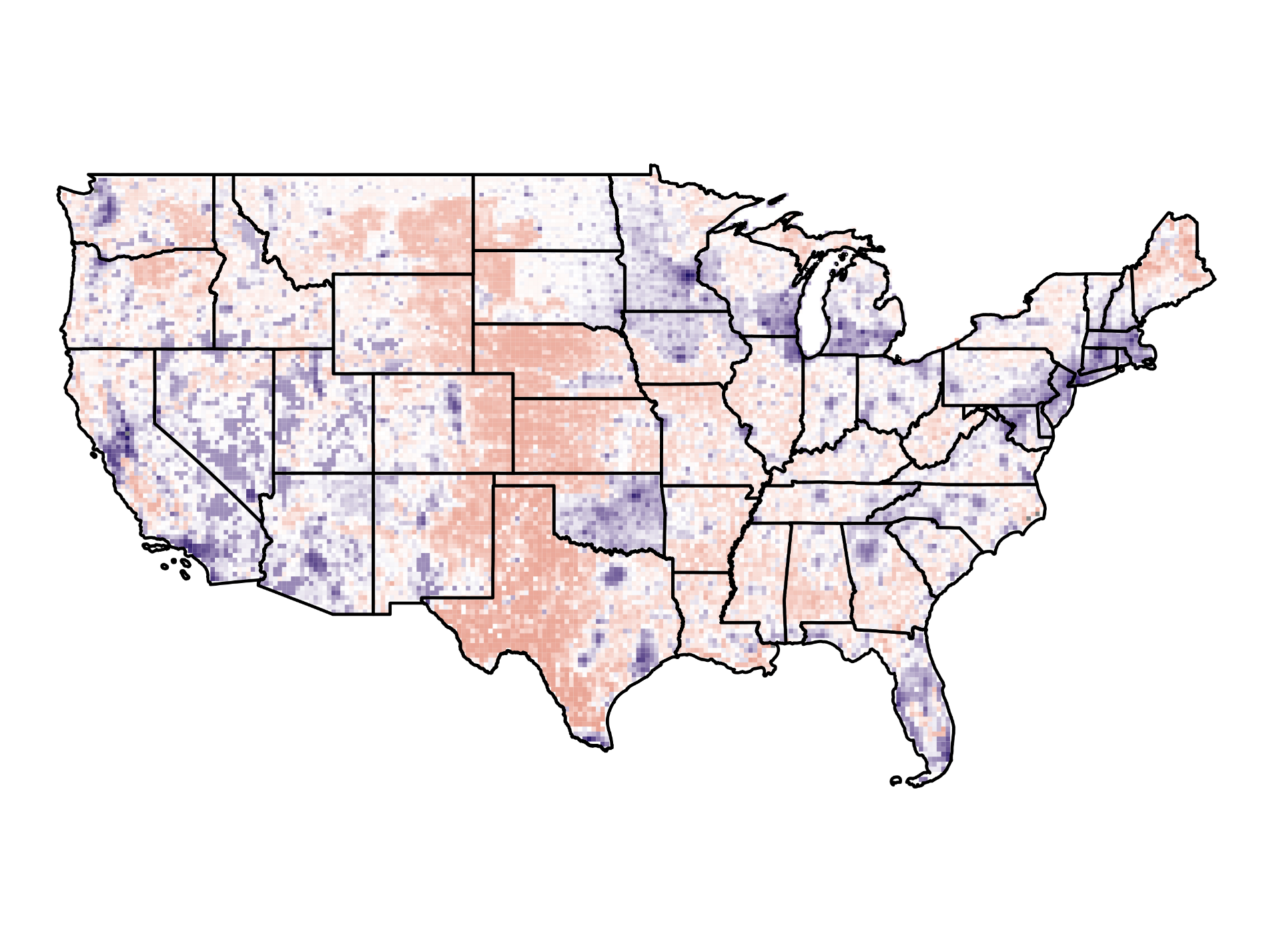


**Supplemental Figure 4.7.** Enlarged predictive map for similarity in Artiodactyla species pool (Jaccard dissimilarity) between iNaturalist and camera trap data. The legend of the color scale is depicted in Figure 3 of the main text.
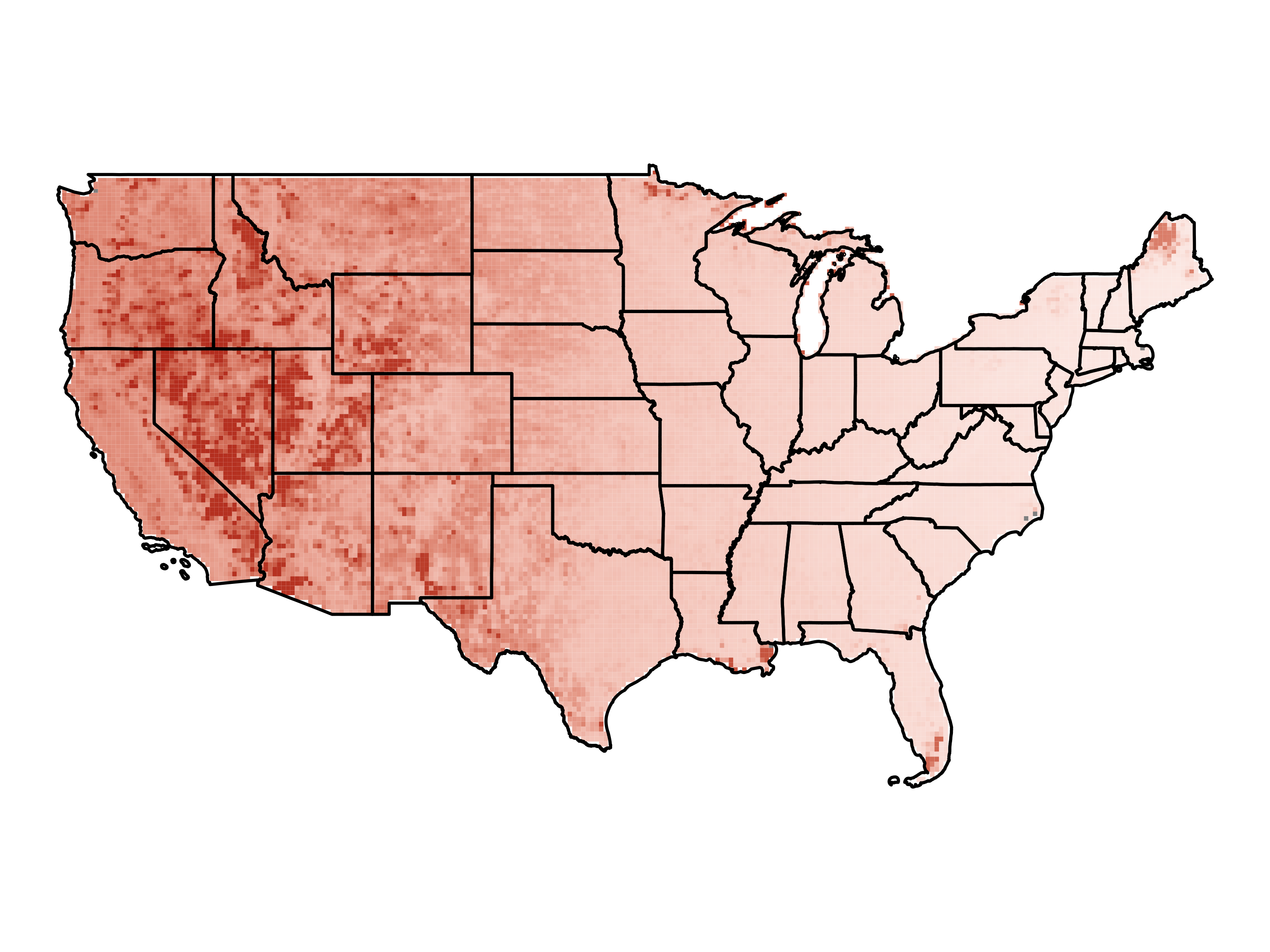


**Supplemental Figure 4.8.** Enlarged predictive map for similarity in Carnivora species pool (Jaccard dissimilarity) between iNaturalist and camera trap data. The legend of the color scale is depicted in Figure 3 of the main text.
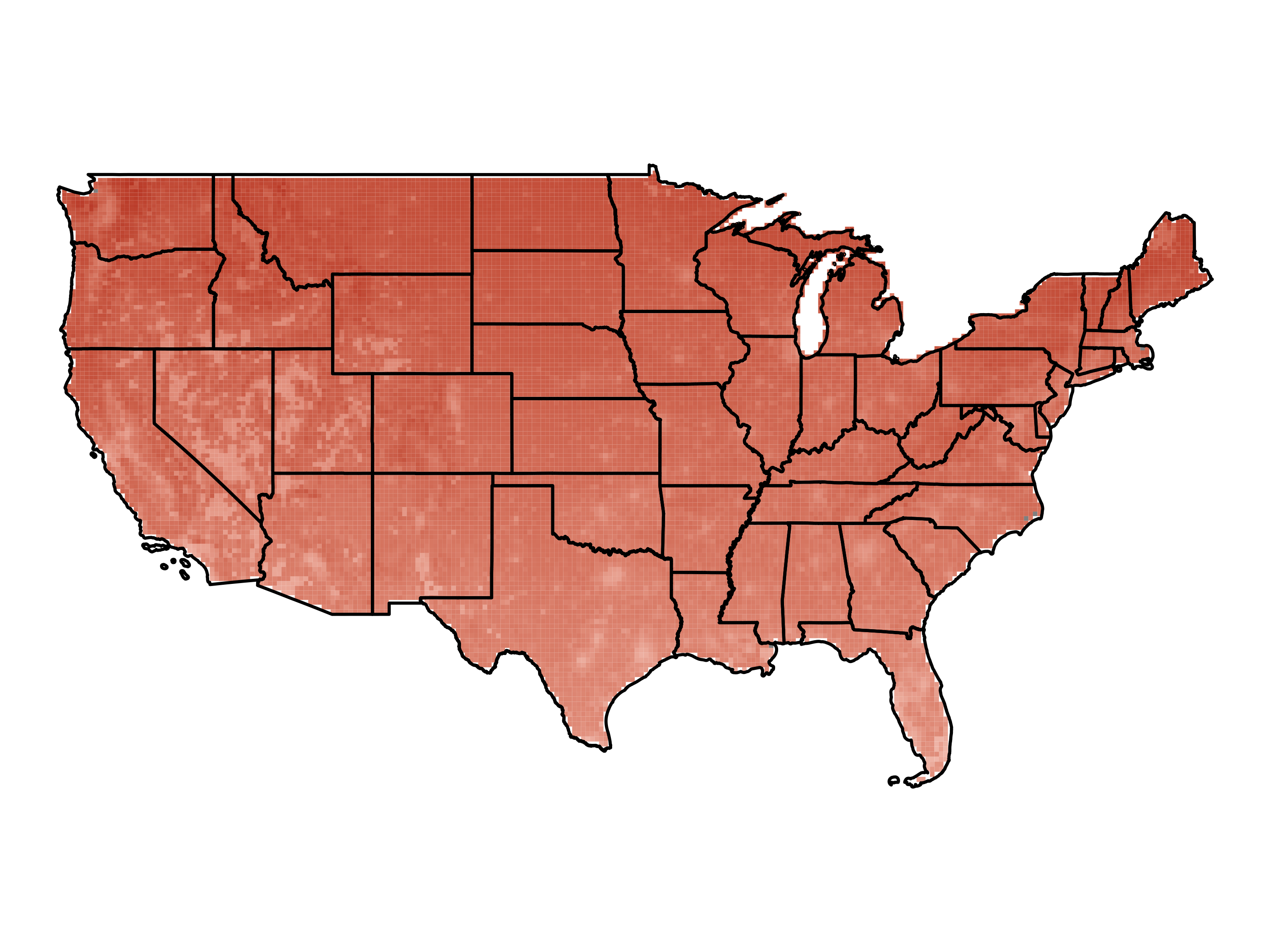


**Supplemental Figure 4.9.** Enlarged predictive map for similarity in Chiroptera species pool (Jaccard dissimilarity) between iNaturalist and camera trap data. The legend of the color scale is depicted in Figure 3 of the main text.
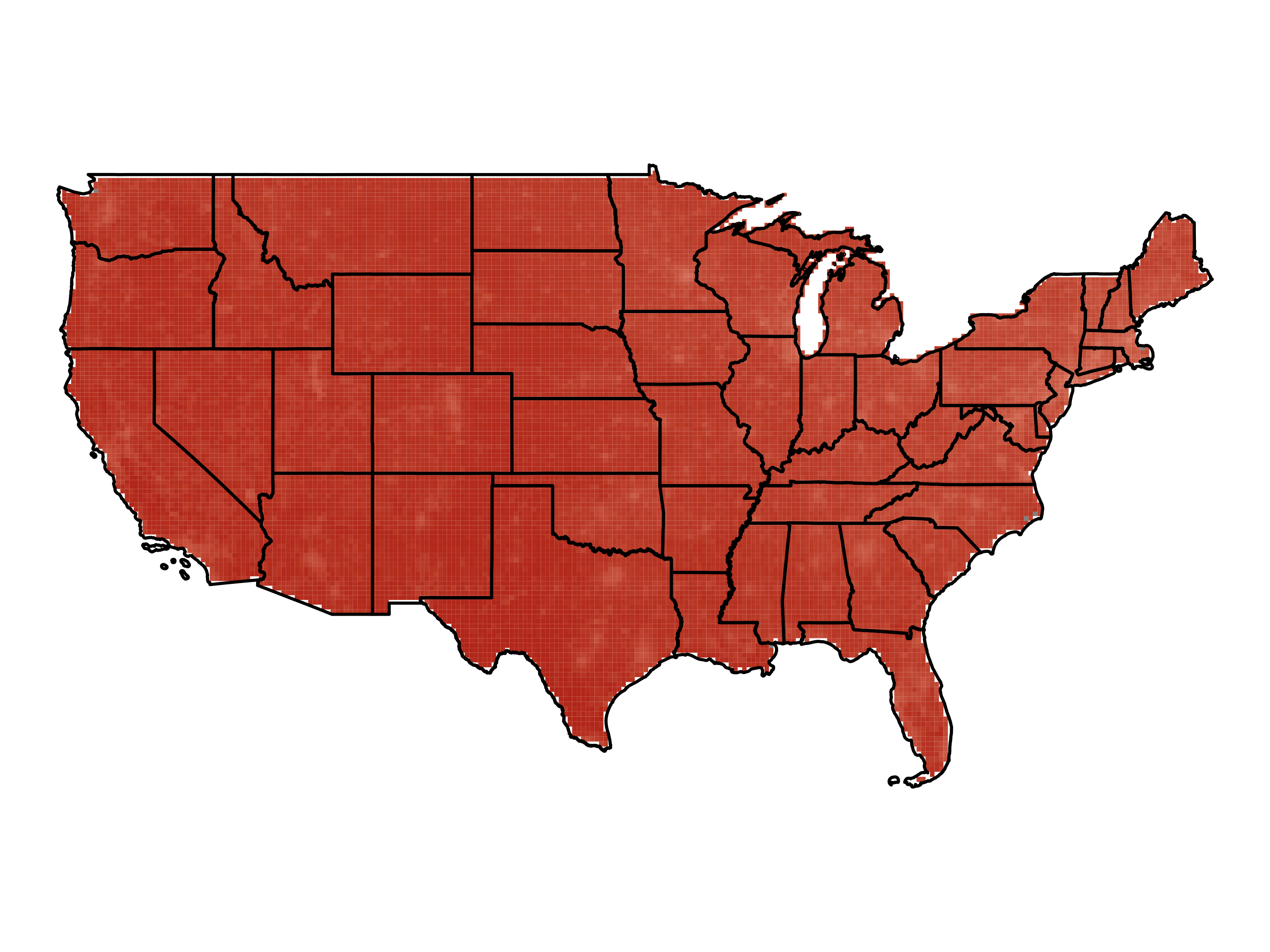


**Supplemental Figure 4.10.** Enlarged predictive map for similarity in Lagomorpha species pool (Jaccard dissimilarity) between iNaturalist and camera trap data. The legend of the color scale is depicted in Figure 3 of the main text.


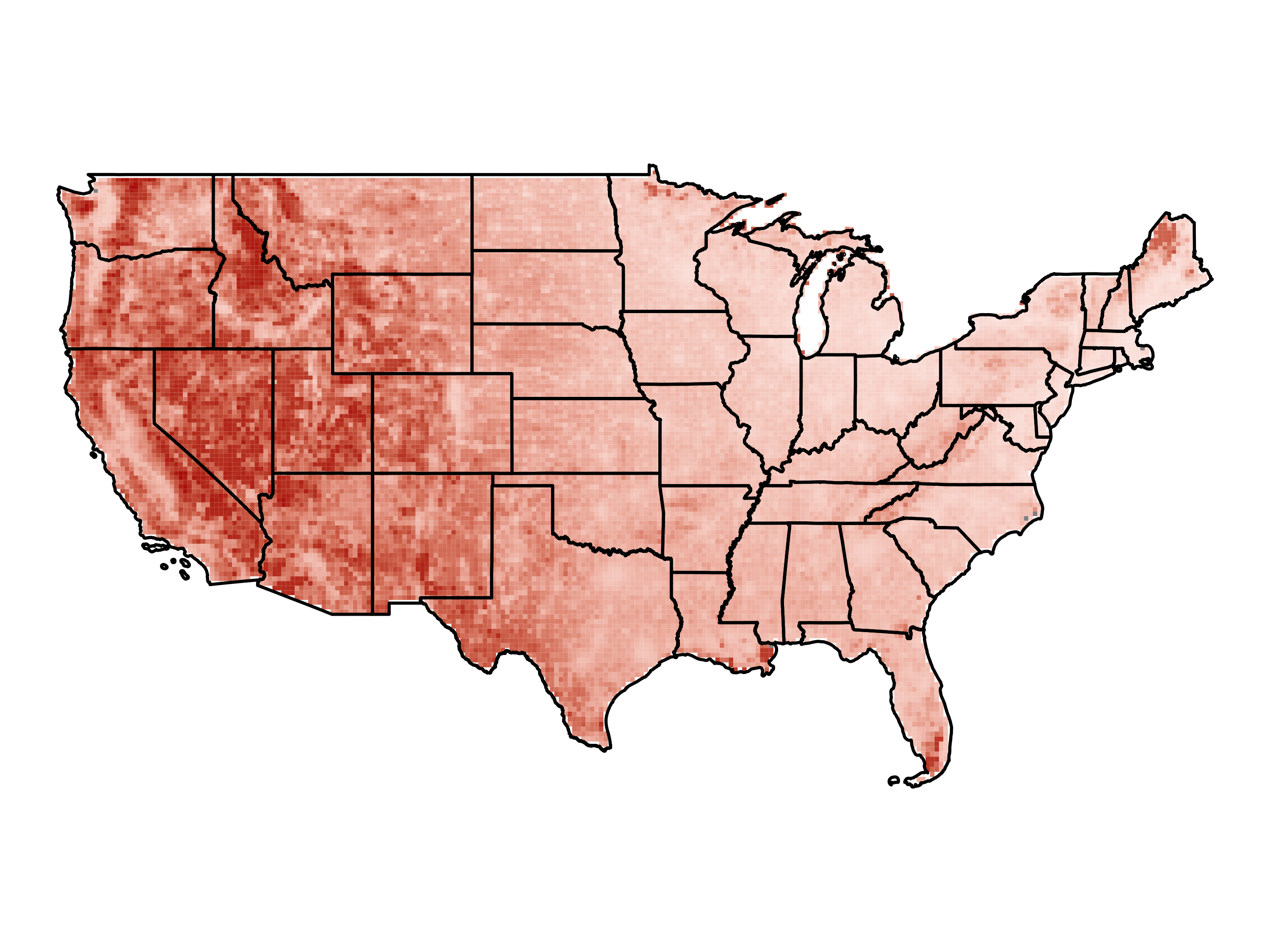


**Supplemental Figure 4.11.** Enlarged predictive map for similarity in Mammalia species pool (Jaccard dissimilarity) between iNaturalist and camera trap data. The legend of the color scale is depicted in Figure 3 of the main text.
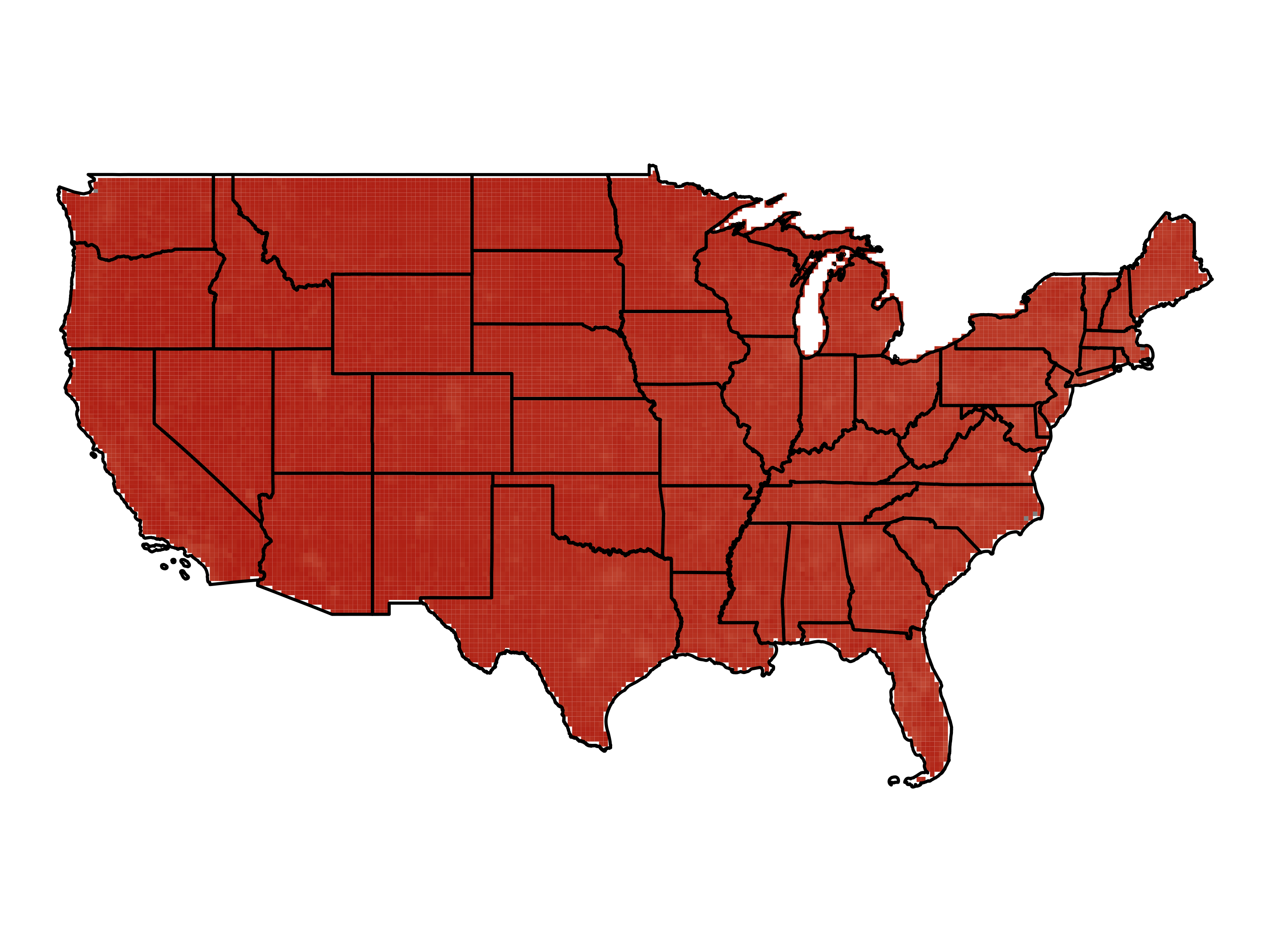


**Supplemental Figure 4.12.** Enlarged predictive map for similarity in Rodentia species pool (Jaccard dissimilarity) between iNaturalist and camera trap data. The legend of the color scale is depicted in Figure 3 of the main text.
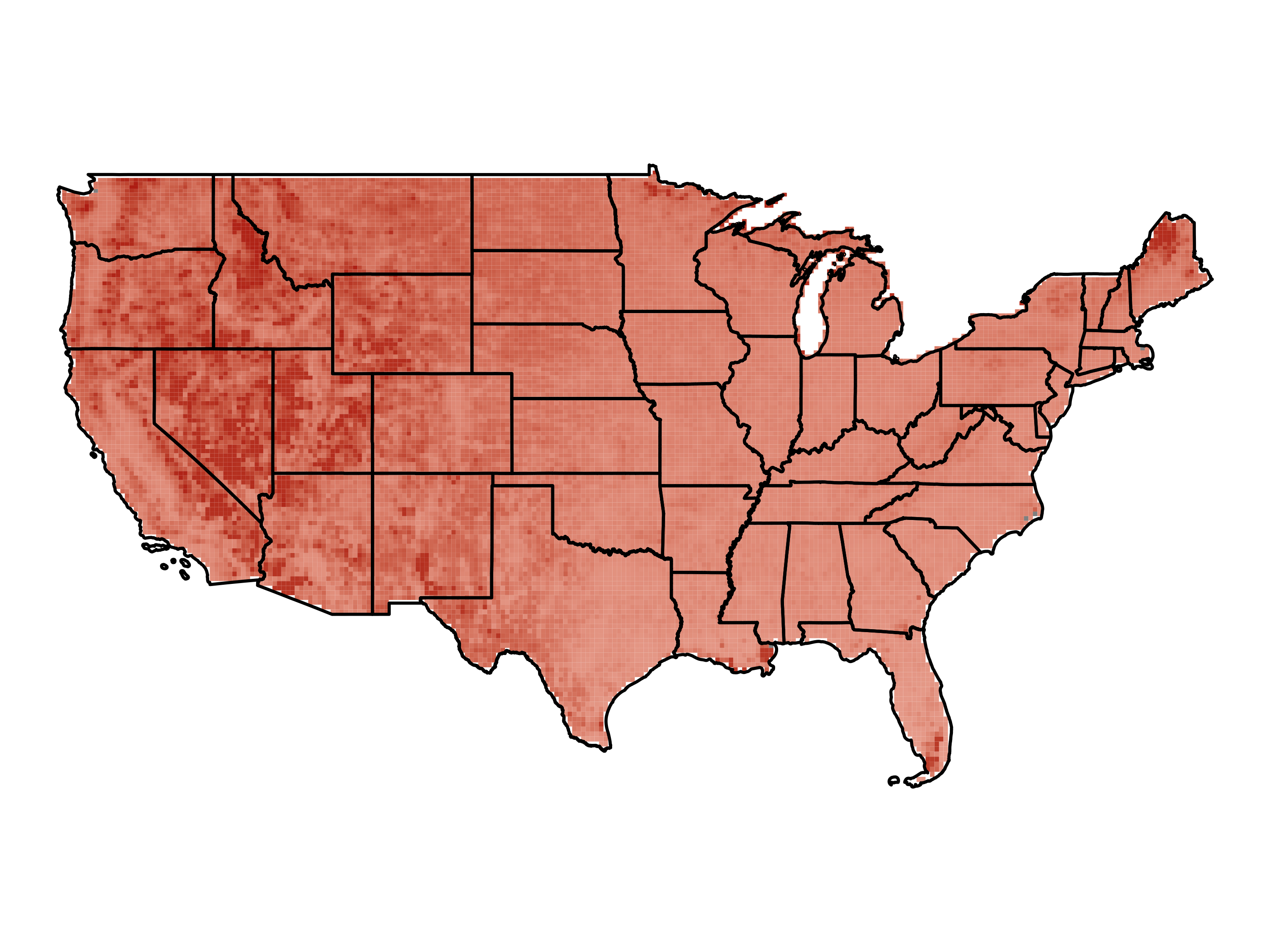

Supplement: Supplementary file 4 — Data S4. [file ECE3-15-e71805-s004.docx]
